# [2.2]Paracyclophane‐Based TCN‐201 Analogs as GluN2A‐Selective NMDA Receptor Antagonists

**DOI:** 10.1002/cmdc.202100400

**Published:** 2021-08-03

**Authors:** Remya Rajan, Dirk Schepmann, Ruben Steigerwald, Julian A. Schreiber, Ehab El‐Awaad, Joachim Jose, Guiscard Seebohm, Bernhard Wünsch

**Affiliations:** ^1^ Institut für Pharmazeutische und Medizinische Chemie der Westfälischen Wilhelms-Universität Münster Corrensstraße 48 48149 Münster Germany; ^2^ Cells-in-Motion Cluster of Excellence (EXC 1003 – CiM) Westfälische Wilhelms-Universität Münster 48149 Münster Germany; ^3^ GRK 2515 Chemical biology of ion channels (Chembion) Westfälische Wilhelms-Universität Münster 48149 Münster Germany; ^4^ Cellular Electrophysiology and Molecular Biology Institute for Genetics of Heart Diseases (IfGH) University Hospital Münster Robert-Koch-Str. 45 48149 Münster Germany

**Keywords:** NMDA receptor, GluN2A subunit, antagonists, TCN-201 analogs, [2.2]paracyclophane, conformational restriction, preorientation, two-electrode voltage clamp

## Abstract

Recent studies have shown the involvement of GluN2A subunit‐containing NMDA receptors in various neurological and pathological disorders. In the X‐ray crystal structure, TCN‐201 (**1**) and analogous pyrazine derivatives **2** and **3** adopt a U‐shape (hairpin) conformation within the binding site formed by the ligand binding domains of the GluN1 and GluN2A subunits. In order to mimic the resulting π/π‐interactions of two aromatic rings in the binding site, a [2.2]paracyclophane system was designed to lock these aromatic rings in a parallel orientation. Acylation of [2.2]paracyclophane (**5**) with oxalyl chloride and chloroacetyl chloride and subsequent transformations led to the oxalamide **7**, triazole **10** and benzamides **12**. The GluN2A inhibitory activities of the paracyclophane derivatives were tested with two‐electrode voltage clamp electrophysiology using *Xenopus laevis* oocytes expressing selectively functional NMDA receptors with GluN2A subunit. The *o*‐iodobenzamide **12 b** with the highest similarity to TCN‐201 showed the highest GuN2A inhibitory activity of this series of compounds. At a concentration of 10 μM, **12 b** reached 36 % of the inhibitory activity of TCN‐201 (**1**). This result indicates that the [2.2]paracyclophane system is well accepted by the TCN‐201 binding site.

## Graphical abstract

**In the binding pocket** of crystallized GluN1/GluN2A ligand binding domain, the prototypical antagonist TCN‐201 and analogs adopt a U‐shaped conformation. [2.2]Paracyclophane‐based GluN2A antagonists were designed to mimic the U‐shaped conformation of TCN‐201 in the binding pocket. The synthesized benzamide shown here possesses 36 % of the GluN2A inhibitory activity of TCN‐201, confirming that compounds with the [2.2]paracyclophane system are well accepted by GluN2A‐NMDA receptors.

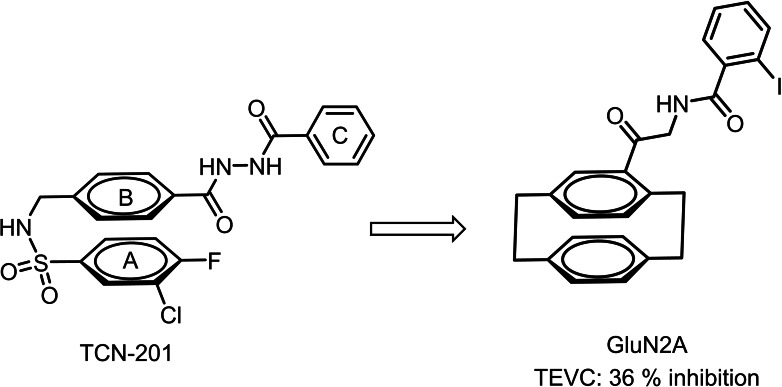

## Introduction

Due to their complex nature, glutamate receptors are among the most interesting targets in the field of medical research. *N*‐Methyl*‐D*‐aspartate (NMDA) receptors belong to the class of ionotropic glutamate receptors. They participate in a variety of physiological processes and are involved in various neurological disorders stimulating interdisciplinary research.^[1]^ NMDA receptors are the only ionotropic glutamate receptors, which require the simultaneous binding of glutamate and glycine at their respective binding sites for activation.[^2^, ^3^] Reaction of the NMDA receptor with glutamate and glycine along with AMPA receptor‐mediated postsynaptic membrane depolarization leads to opening of the receptor‐associated ion‐channel allowing Ca^2+^ ions to permeate into the neuron. Elevated intracellular Ca^2+^ ion concentrations are required for long‐term potentiation (LTP) and long‐term depression (LTD), processes essential for synaptic plasticity and memory function. On the other hand, strong NMDA receptor stimulation can cause excitotoxicity.[^4^, ^5^] Abnormal NMDA receptor activation resulting in very high intracellular Ca^2+^ ion concentration is associated with several pathological neurological conditions like stroke, status epilepticus as well as Parkinson's, Alzheimer's and Huntington's disease.^[6]^ Prevention of this receptor overactivation and subsequent reduction of resulting excitotoxicity may be therapeutically beneficial for the treatment of these disorders.

Four subunits are required to form the active heterotetrameric NMDA receptor. Each receptor subunit consists of four domains: the extracellular amino‐terminal domain (ATD), the ligand binding domain (LBD), the transmembrane domain (TMD) built up by three transmembrane helices and a re‐entrant loop embedded in the cell membrane, and finally the intracellularly located carboxy‐terminal domain (CTD). The size of the intracellular CTD varies depending on the subunit type. It has multiple sites for interaction with intracellular proteins anchoring the NMDA receptor at the cytoskeleton.^[7]^ The high variability of the NMDA receptor results from the large number of subunits forming the ion channel receptor. Seven subunits termed GluN1, GluN2A‐D and GluN3A‐B subunits encoded by seven separate genes (GRIN1, GRIN2A‐D and GRIN3A‐B) are known to form the NMDA receptor. The complexity of the NMDA receptor is even increased by the existence of eight different splice variants of the GluN1 subunit termed GluN1a‐h due to the alternate splicing.[^8^, ^9^, ^10^] The existence of a large number of binding sites, e. g., for endogenous agonists glycine (GluN1 subunit) and (*S*)‐glutamate (GluN2 subunit), exogenous phencyclidine like channel blockers and Mg^2+^ ions (channel pore), polyamines, Zn^2+^ ions and protons (ATD) considerably increases the complex fine‐tuned modulation of the NMDA receptor activation.^]11,12]^

The gating properties of the NMDA receptor are mainly associated with the type of GluN2 subunit present in the heterotetrameric ion channel. NMDA receptors with GluN2C and GluN2D subunits are less Ca^2+^ conductive than NMDA receptors with GluN2A and GluN2B subunits. GluN2A subunit containing NMDA receptors open and close faster than the corresponding NMDA receptors with GluN2B subunit. The high sensitivity of GluN2A subunit containing NMDA receptors to (*S*)‐glutamate and glycine, the fast activation and deactivation kinetics[^13^, ^14^, ^15^] and the developmental switch of GluN2A to GluN2B subunits represent important characteristics of GluN2A‐NMDA receptors rendering them unique among the different NMDA receptor subtypes.[^16^, ^17^]

The expression of the GluN2A subunit in the brain is found to be high in the hippocampus and the cerebral cortex. Moderate concentrations of the GluN2A subunit are expressed in the midbrain, cerebellum, striatum, and brainstem, whereas the olfactory bulb and hypothalamus exhibit only low expression of the GluN2A subunit. In the periphery, the GluN2A subunit has been found in the heart, where it is restricted to the atria,^[18]^ glomerular cells in the kidney,^[19]^ and mouse bone marrow cells.^{20]^ Moreover, overexpression of the GluN2A subunit was found in pancreatic cancer cells.^[21]^

Since appropriate GluN2A selective ligands are missing, the exact role of GluN2A subunit‐containing NMDA receptors remains unclear. Therefore, the synthesis and biological evaluation of negative allosteric modulators (NAMs) are envisaged in this article.

**Figure 1:**
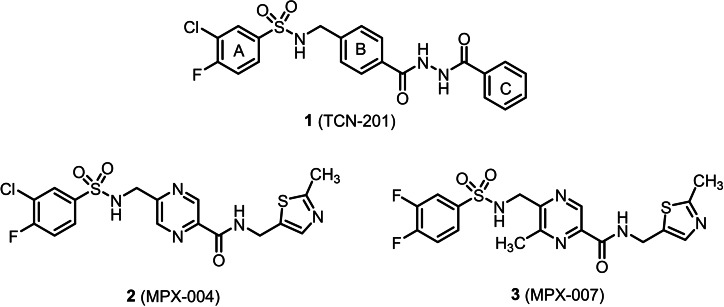
NAMs selectively inhibiting GluN2A subunit containing NMDA receptors.

TCN‐201 (**1**) represents the prototypical NAM for GluN2A subunit‐containing NMDA receptors. (Figure 1) It interacts with a binding site at the interface between the LBDs of the GluN1 and GluN2A subunits, which leads to a conformational change of the receptor and finally prevents the binding of glycine to the glycine binding site on the GluN1 subunit. Patch clamp experiments using GluN1/GluN2A transfected HEK293T cells revealed an IC_50_ value of 109 nM for TCN‐201.[^22^, ^23^] Even though TCN‐201 (**1**) showed ion flux inhibition in the nanomolar range, its inhibitory activity decreased with increasing concentrations of glycine with almost no inhibition at a glycine concentration of 300 μM. Another drawback of TCN‐201 (**1**) is its poor solubility. Replacement of the ring in the middle of TCN‐201 by a pyrazine ring led to MPX‐004 (**2**) and MPX‐007 (**3**) with improved solubility. Even though MPX‐004 (**2**) and MPX‐007 (**3**) showed higher inhibitory activity at GluN2A NMDA receptors (IC_50_ (**2**)=79 nM, IC_50_ (**3**)=27 nM) than TCN‐201, the inhibitory activity of **2** and **3** was also reduced by high concentrations of glycine.^[24]^ (Figure 1) Replacement of the benzene ring in the middle of TCN‐201 by electron‐rich five‐membered aromatic heterocycles such as thiazole, oxazole and isoxazole rings led to reduced GluN2A NMDA inhibition.^[25]^ However, systematic modifications of the benzenesulfonamide^]26]^ and the benzoylhydrazine moiety^]27]^ of TCN‐201 could slightly improve the inhibitory activity at GluN2A containing NMDA receptors.

**Figure 2:**
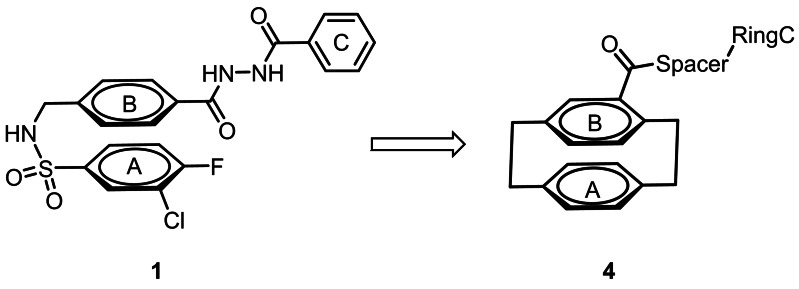
Selective GluN2A subunit containing NMDA receptor NAM designed by replacing ring A and ring B by the [2.2]paracyclophane system.

The X‐ray crystal structure (PDB 5I56) consisting of one GluN1 and one GluN2A LBD in complex with TCN‐201 (**1**) shows a U‐shaped or hairpin like conformation of TCN‐201 within the binding pocket. The halogenated aromatic A ring of **1** forms a sandwich with the aromatic benzene ring in the middle (B ring) resulting in a parallel orientation of these rings stabilized by π‐π interactions.^[28]^ (Figure 2) A similar orientation was found for MPX‐004 (**2**; PDB 5I58) and MPX‐007 (**3**; PDB 5I58) within the same binding site.^[29]^

In order to pre‐orientate the two aromatic rings in a parallel fashion and thus increase the π‐π interactions between the aromatic rings, a [2.2]paracyclophane system should replace the aromatic rings A and B of TCN‐201 (see compound **4** in Figure 2). Herein, we report on the synthesis and biological evaluation of [2.2]paracyclophane‐based TCN‐201 analogs of type **4**, which have been designed to confirm this unusual U‐shaped conformation of NAMs in the binding pocket of GluN2A NMDA receptors.

## Results and Discussion

### Synthesis

**Scheme 1:**
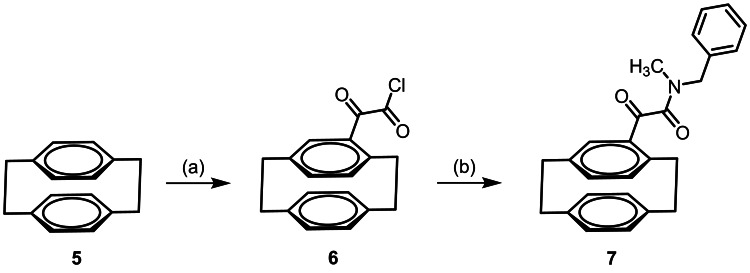
Synthesis of racemic oxalyl derivative **7**. Reagents and reaction conditions: (a) Oxalyl chloride, AlCl_3_, CH_2_Cl_2_, 0 °C to rt, 4 h, 89 %. (b) *N*‐Methylbenzylamine, Et_3_N, 0 °C to rt, 30 min, 24 %.

The synthesis of paracyclophane‐based TCN‐201 analogs of type **4** started with [2.2]paracyclophane (**5**) consisting of two co‐facially stacked and strongly interacting benzene rings connected at the *p*‐positions by two ethylene bridges. Friedel‐Crafts acylation of [2.2]paracyclophane (**5**) with oxalyl chloride and AlCl_3_ led to the monosubstituted racemic paracyclophane **6** bearing the substituent at the aromatic ring.^[30]^ Reaction of the acid chloride **6** with *N*‐methylbenzylamine provided the amide **7**. (Scheme 1)

**Scheme 2:**
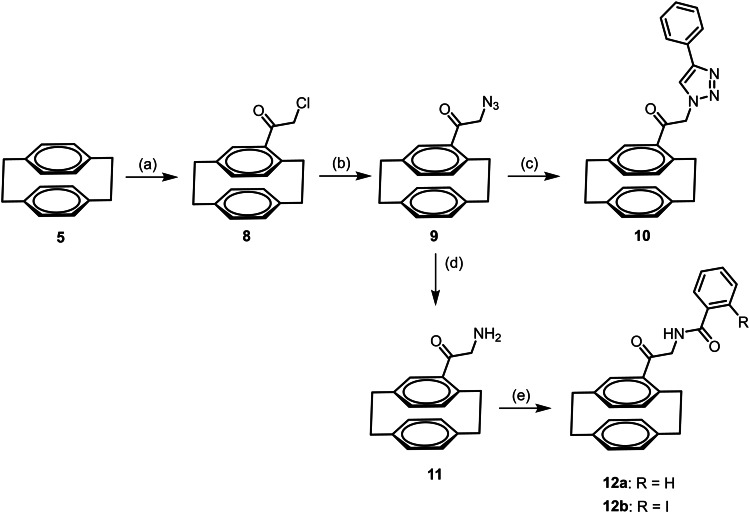
Synthesis of racemic [2.2]paracyclophane‐based triazole **10** and benzamides **12**. Reagents and reaction conditions: (a) Chloroacetyl chloride, AlCl_3_, CH_2_Cl_2_, 0 °C to rt, 4 h, 85 %. (b) NaN_3_, KI, DMF, rt, 10 h, 99 %. (c) Phenylacetylene, CuSO_4_^.^5H_2_O, sodium ascorbate, water, rt, 10 min, 86 %. (d) H_2_, Pd/C, THF:MeOH 1 : 1, rt, 16 h, 86 %. (e) R−C_6_H_4_CO_2_H, COMU, DIPEA, THF, rt, 16 h, **12 a**: 88 %; **12 b**: 84 %.

Alternatively, [2.2]paracyclophane (**5**) was acylated with 2‐chloroacetyl chloride and AlCl_3_ to afford the chloroacetamide **8** in 85 % yield. Nucleophilic substitution of the α‐chloro ketone **8** with NaN_3_ provided the α‐azido ketone **9** in 99 % yield, which reacted in a Cu(I) catalyzed Huisgen 1,3‐dipolar cycloaddition with phenylacetylene to give the racemic 1,4‐disubstituted triazole **10** in 86 % yield. (Scheme 2)

The Pd‐catalyzed hydrogenation of the α‐azido ketone **9** led to the α‐amino ketone **11**, which was acylated with benzoic acid and 2‐iodobenzoic acid to afford the racemic benzamides **12 a** and **12 b** in 88 and 84 % yield, respectively. (Scheme 2)

### Pharmacological evaluation

The antagonistic activity of the [2.2]paracyclophane derivatives was tested by two‐electrode voltage clamp (TEVC) using *Xenopus laevis* oocytes as previously described.[^25^, ^26^, ^27^] cRNAs for the GluN1a and GluN2A subunits were injected into defolliculated oocytes and the oocytes were incubated at 16 °C for 4 days. The membrane current due to NMDA receptor activation, which was achieved with 10 μM (*S*)‐glutamate and 10 μM glycine, was recorded. After addition of the test compounds, the changed membrane currents were recorded and normalized to the inhibition of the reference compound TCN‐201 (=100 %).

**Figure 3:**
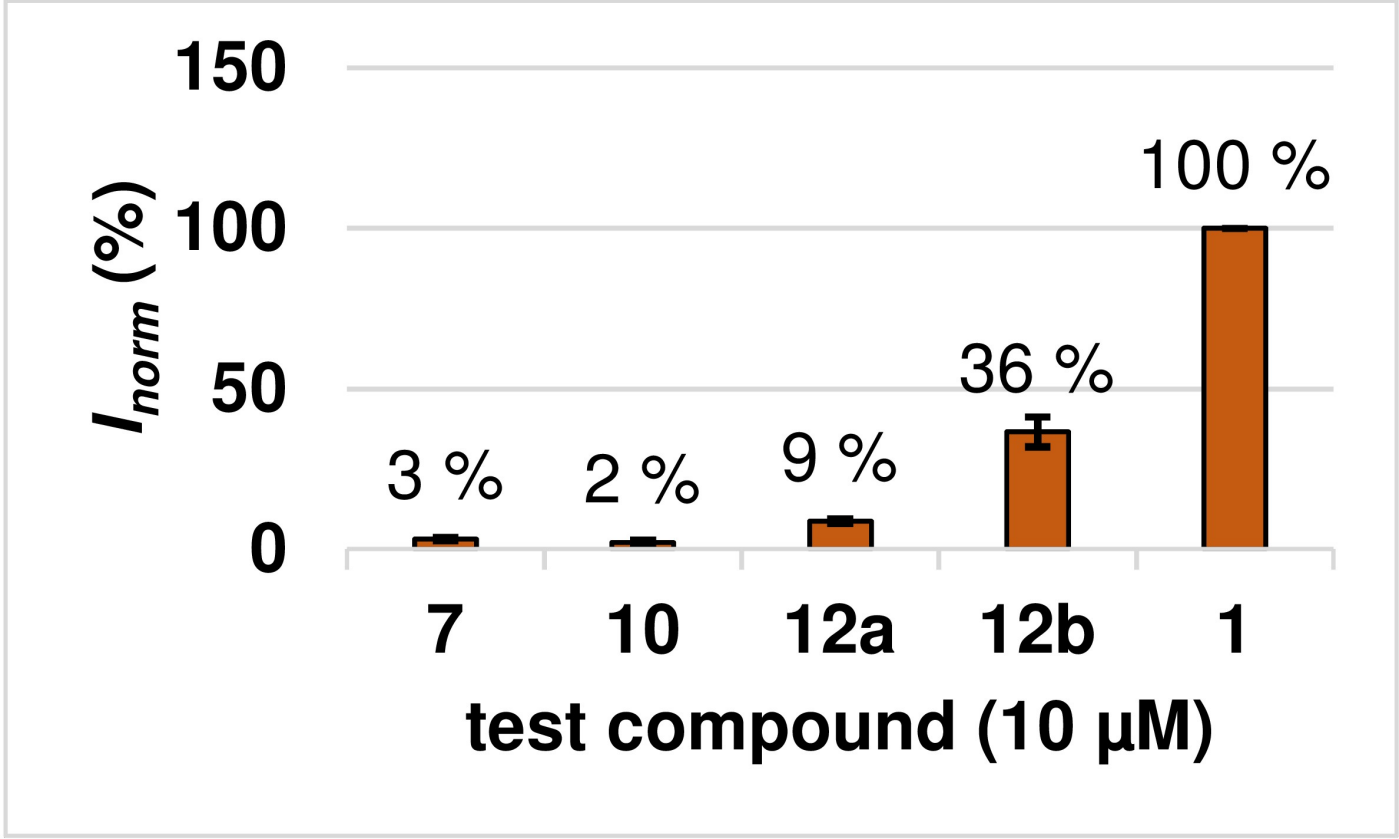
Normalized inhibition (*I_norm_*) of test compounds with paracyclophane scaffold. The reduced ion flux across the oocyte membrane was measured at a concentration of 10 μM of the test compounds after activation with 10 μM (*S*)‐glutamate and 10 μM glycine. The inhibition obtained by TCN‐201 (**1**, 10 μM) was set to 100 % and the inhibition of the test compounds (10 μM) was normalized to this activity (in %). The activity of each compound was measured with three independent oocytes (n=3).

At a concentration of 10 μM, the [2.2]paracyclophane‐based 2‐oxoamide **7** exhibited 3 % of the inhibition of TCN‐201 (**1**) (Figure 3). The triazole **10** showed the same activity as the oxoamide **7**. Although a slight increase of the normalized GluN2A channel inhibition was observed for the benzamide **12 a** (*I_norm_* =9 %), this value is still in the “blind region” of the TEVC setup (<10 % inhibition). However, the analogous o‐iodobenzamide **12 b** showed 36 % of the inhibition of TCN‐201. The comparably high GluN2A NMDA receptor inhibition of the benzamides **12** is explained by their structural similarity with the U‐shaped structure of TCN‐201 (**1**). The benzamides **12** result from replacement of an NH‐moiety of TCN‐201 (**1**) by a methylene moiety.

The [2.2]paracyclophane‐based compounds **7**, **10** and **12** were also analyzed for their affinity towards the ifenprodil binding site of GluN2B NMDA receptors,[^31^, ^32^] σ_1_ and σ_2_ receptors.[^33^, ^34^, ^35^] At a concentration of 10 μM, the [2.2]paracyclophanes did not interact with the ifenprodil binding site of GluN2B NMDA receptors, σ_1_ and σ_2_ receptors. These results indicate the selectivity of the benzamides **12** for NMDA receptors containing the GluN2A subunit over those NMDA receptors containing the GluN2B subunit, as well as over σ_1_ and σ_2_ receptors.

## Conclusion

[2.2]Paracyclophane‐based GluN2A receptor ligands were designed to imitate the U‐shaped structure of TCN‐201 (**1**) and analogous pyrazine derivatives **2** and **3** in the receptor binding pocket. Although the benzamides **12 a** and **12 b**, which are structurally most similar to the lead compounds, do not reach the inhibitory activity of TCN‐201, they show considerable inhibition of GluN2A subunit‐containing NMDA receptors. The promising inhibitory activity of **12 b** indicates that the [2.2]paracyclophane system is well accepted by the binding site of the GluN2A NMDA receptor.

## Experimental Section

### Chemistry, general

Moisture and oxygen sensitive reactions were carried out under nitrogen in dry glassware (Schlenk tubes or flasks). Reaction mixtures were stirred with magnetic stirrer MR 3001 K (Heidolph). As source of ultra‐sonication an ultrasonic bath Sonorex Super (Bandelin electronic GmbH, Germany) was used. Temperatures were controlled with dry ice/H_2_O (0 °C), magnetic stirrer MR 3001 K (Heidolph), together with temperature controller EKT HeiCon (Heidolph) or VT‐5 (VWR) and PEG or silicone bath. Chemical structures were generated by ChemDraw Professional 16.0 (v16.0.1.4 (77)). All solvents were of analytical grade quality. Demineralized water was used. Water free solvents were freshly distilled under N_2_ atmosphere or stored over molecular sieves prior to use; CH_2_Cl_2_: Distilled from calcium hydride; THF: Distilled from sodium/benzophenone; Methanol: Distilled from magnesium methanolate; Dimethyl sulfoxide, dimethylformamide and toluene: Stored over molecular sieves 4 Å. Thin layer chromatography (TLC) was conducted with TLC silica gel 60 F254 on aluminum sheets (Merck) as stationary phase in a saturated chamber at room temperature. Spots were visualized with UV light (254 nm or 366 nm). Additionally, TLC plates were dipped in suitable staining baths to make compounds visible. Compositions of mobile phase and retention factors (R_f_) of compounds are given in the compound descriptions. Flash chromatography (fc): Silica gel 60, 40–63 μm (VWR); parentheses include: diameter of the column (Ø), length of the stationary phase (l), fraction size (v) and eluent. Automated flash chromatography: Isolera^TM^ Spektra One (Biotage®); parentheses include: cartridge size, flow rate, eluent, fractions size was always 20 mL. Melting point: Melting point system MP50 (Mettler Toledo, Gießen, Germany), open capillary, uncorrected. Mass spectrometry (MS): All samples were measured in a positive ion method. Exact mass spectra of synthesized compounds were recorded as follows: Exact Mass (APCI): Atmospheric pressure chemical ionization (APCI) mass spectra were recorded with a MicroTOFQII mass spectrometer (Bruker Daltonics). The deviations of the found exact mass from the calculated exact masses were 5 mDa or less unless otherwise stated. The data were analyzed with DataAnalysis (Bruker Compass 4.1). NMR spectra were recorded on Agilent DD2 400 MHz and 600 MHz spectrometers. The frequencies are given in the descriptions of the synthetic procedures. MestReNova software (version 11.0.1‐17801, © 2016 by Mestrelab Research S.L.) was used for analyzing NMR spectra. Chemical shifts (δ) are reported in parts per million (ppm) against the reference substance tetramethylsilane (TMS) and calculated using the solvent residual peak of the deuterated solvent. Infrared (IR) spectra were obtained on a FTIR Prestige 21 (Shimadzu) using attenuated total reflection (ATR) technique. All samples were applied to the device without solvent and were directly measured. Absorption bands are characterized by their wave numbers ṽ [cm^−1^].

### High‐performance liquid chromatography (HPLC)

HPLC was used to determine the purity of the synthesized compounds. Equipment: pump: L‐6200A; UV detector: L‐7400; data acquisition: HSM‐Software (all from Merck Hitachi); Column: phenomenexGemini® 5 μm C6‐Phenyl 110 Å, LC Column 250×4.6 mm; Solvents: A: demineralized water with 0.05 % (*v*/*v*) trifluoroacetic acid B: acetonitrile with 0.05 % (*v*/*v*) trifluoroacetic acid; gradient elution (% A): 0–4 min: 90 %; 4–29 min: gradient from 90 % to 0 %; 29–31 min: 0 %; 31–31.5 min: gradient from 0 % to 90 %; 31.5–40 min: 90 %. Flow rate: 1.0 mL/min, Injection volume: 5.0 μL, wavelength: 210 nm.

### Synthetic procedures

The starting materials were purchased from different commercial sources and were of analytical grade. The synthesis of the intermediate **6** was already reported in literature,^[30]^ but optimized within this project.

### (±)‐2‐Oxo‐2‐([2.2]paracyclophan‐4‐yl)ethanoyl chloride (6)^[30]^


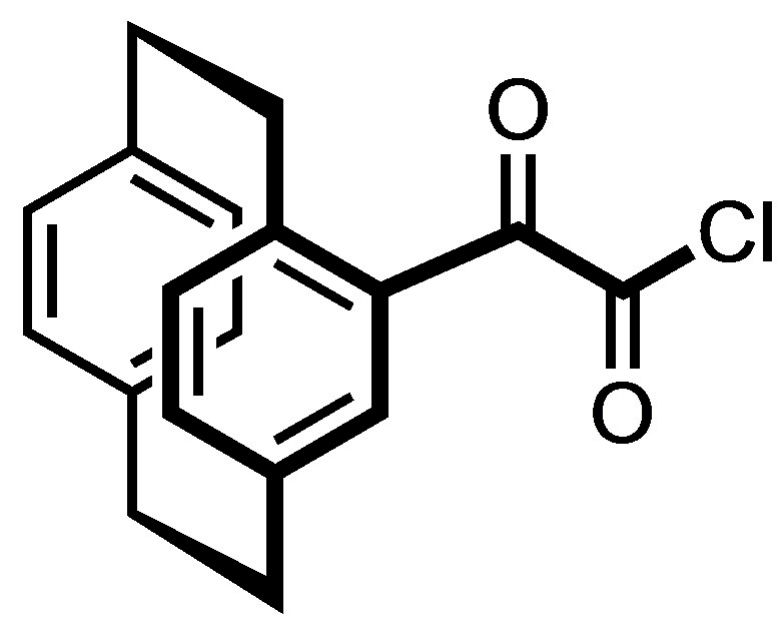


Paracyclophane (**5**, 1.0 g, 4.8 mmol, 1.0 eq.) was dissolved in CH_2_Cl_2_ (30 mL) and the solution was cooled to 0 °C. Oxalyl chloride (1.1 mL, 12.9 mmol, 2.7 eq.) was added to a solution of AlCl_3_ (1.4 g, 10.6 mmol, 2.2 eq.) in CH_2_Cl_2_ (12 mL) and this solution was added dropwise to the solution of **5** at 0 °C. The reaction mixture was warmed to room temperature and stirred for 4 h. After completion of the transformation, saturated Na_2_CO_3_ solution (20 mL) was added to the reaction mixture. The organic layer was separated, and the aqueous layer was extracted with CH_2_Cl_2_ (3×30 mL). The organic layer was dried (Na_2_SO_4_) and concentrated in vacuo. The residue was obtained as a light brown oil that was proceeded for the next step without further purification. Light brown oil, yield 1.28 g (89 %). R_f_=0.74 (cyclohexane/ethyl acetate 9 : 1). C_18_H_15_ClO_2_ (298.8 g/mol).

### (±)‐N‐Benzyl‐N‐methyl‐2‐oxo‐2‐([2.2]paracyclophan‐4‐yl)ethanamide (7)


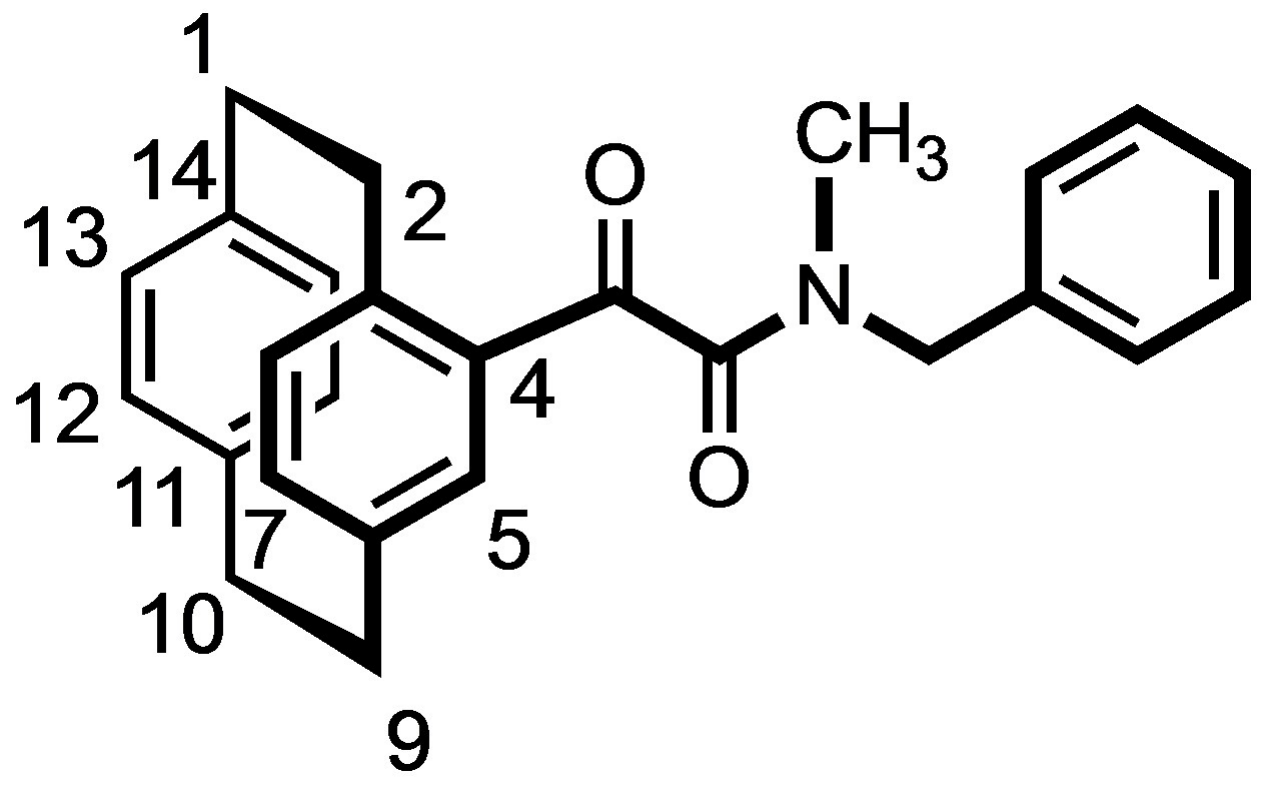


Acid chloride **6** (1.00 g, 3.3 mmol, 1.0 eq.) was dissolved in CH_2_Cl_2_ (40 mL), and Et_3_N (1.36 mL, 9.9 mmol, 3.0 eq.) was added. The solution was cooled to 0 °C and *N*‐methylbenzylamine (0.39 mL, 3.0 mmol, 0.9 eq.) was added after 10 min. The reaction mixture was warmed to rt. After stirring for 30 min, 10 M HCl (40 mL) was added to the reaction mixture. The organic layer was separated, and the aqueous layer was extracted with CH_2_Cl_2_ (3×40 mL). The organic layer was dried (Na_2_SO_4_) and concentrated in vacuo. The residue was purified by FCC (Ø=4 cm, l=25 cm, V=75 mL, cyclohexane/ethyl acetate 92 : 8. R_f_=0.33 (cyclohexane/ethyl acetate 9 : 1). Colorless oil, yield 0.33 g (24 %). C_26_H_25_NO_2_ (383.5 g/mol). ^1^H NMR (400 MHz, CD_3_OD): δ (ppm)=2.57 (s, 3×0.55H, C*H_3_*), 2.90–3.18 (m, 3×0.45H, C*H_3_* *, 8×0.55H, C*H*_*2*(paracyclophane)_, 8×0.45H, C*H*_*2*(paracyclophane)_*), 4.08 (d, *J*=15.5 Hz, 0.45H, NC*H_2_* Ph*), 4.29 (d, *J*=15.5 Hz, 0.45H, NC*H_2_* Ph*), 4.61 (d, *J*=14.4 Hz, 0.55H, NC*H_2_* Ph), 4.84 (d, *J*=14.5 Hz, 0.55H, NC*H_2_* Ph), 6.36 (dd, *J*=7.8/1.9 Hz, 0.45H, 12‐H*), 6.39 (dd, *J*=7.8/1.9 Hz, 0.55H, 12‐H), 6.46 (dd, *J*=7.8/3.1 Hz, 1H, 13‐H), 6.51 (d, *J*=2.1 Hz, 1H, 5‐H), 6.53–6.58 (m, 2H, 15‐H, 16‐H), 6.57–6.60 (m, 1H, 7‐H), 6.92 (d, *J*=7.5 Hz, 2×0.45H, 2‐H_phenyl_*, 6‐H_phenyl_*), 6.95 (dd, *J*=7.8/1.9 Hz, 0.55H, 8‐H), 7.01 (dd, *J*=7.9/1.9 Hz, 0.45H, 8‐H*), 7.16–7.23 (m, 3×0.45H, 3‐H_phenyl_*, 4‐H_phenyl_*, 5‐H_phenyl_*), 7.28–7.32 (m, 0.55H, 4‐H_phenyl_), 7.35–7.39 (m, 4×0.55H, 2‐H_phenyl_, 3‐H_phenyl_, 5‐H_phenyl_, 6‐H_phenyl_). Ratio of rotamers 55 : 45; signals of the minor rotamer are marked with asterisks. ^13^C NMR (151 MHz, CD_3_OD): δ (ppm)=34.4 (0.45 C, N(*C*H_3_)CH_2_*), 35.7 (0.55 C, C‐10), 35.9 (0.45 C, C‐10*), 37.2 (0.45 C, C‐1*), 37.3 (0.55 C, C‐1), 37.3 (0.45 C, C‐9*), 37.4 (0.55 C, C‐9), 37.4 (0.55 C, C‐2) 37.5 (0.45 C, C‐2*), 37.7 (0.55 C, N(*C*H_3_)CH_2_), 52.8 (0.45 C, N*C*H_2_Ph*), 56.3 (0.55 C, N*C*H_2_Ph), 129.4 (2×0.45 C, C‐2_phenyl_*, C‐6_phenyl_*), 129.8 (0.45 C, C‐4_phenyl_*), 129.9 (0.55 C, C‐4_phenyl_) 130.4 (2×0.55 C, C‐2_phenyl_, C‐6_phenyl_), 130.9 (2×0.55 C, C‐3_phenyl_, C‐5_phenyl_), 131.0 (2×0.45 C, C‐3_phenyl_*, C‐5_phenyl_*), 132.9 (0.45 C, C‐5*), 133.1 (0.55 C, C‐5), 134.3 (0.45 C, C‐8*), 134.4 (0.55 C, C‐8), 134.7 (0.55 C, C‐7), 134.8 (0.45 C, C‐7*), 134.9 (1 C, C‐12), 135.0 (0.45 C, C‐3*), 135.2 (0.55 C, C‐3), 135.3 (0.45 C, C‐13*), 135.4 (0.55 C, C‐13), 137.1 (0.45 C, C‐15*), 137.2 (0.55 C, C‐15), 137.5 (0.45 C, C‐16*), 137.6 (0.55 C, C‐16), 139.2 (0.45 C, C‐1_phenyl_*) 139.8 (0.55 C, C‐1_phenyl_), 140.1 (1 C, C‐14), 141.6 (0.55 C, C‐11), 141.7 (0.45 C, C‐11*), 141.8 (0.45 C, C‐6*), 141.9 (0.55 C, C‐6), 142.8 (0.55 C, C‐4), 142.8 (0.45 C, C‐4*), 174.4 (0.55 C, *C*(=O)N), 175.1 (0.45 C, *C*(=O)N*), 193.9 (C=O*), 195.2 (C=O). Signals of the minor rotamer are marked with asterisks.

### (±)‐2‐Chloro‐1‐([2.2]paracyclophan‐4‐yl)ethan‐1‐one (8)


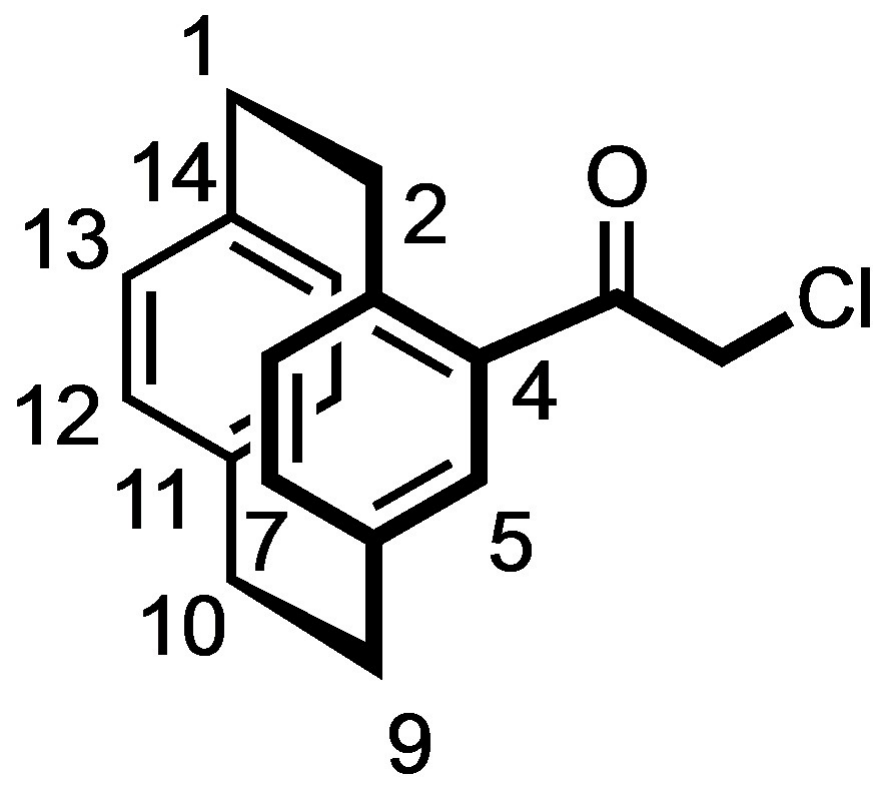


[2.2]Paracyclophane (**5**, 0.50 g, 2.4 mmol, 1.0 eq.) in CH_2_Cl_2_ (15 mL) was cooled to 0 °C. Chloroacetyl chloride (0.52 mL, 6.5 mmol, 2.7 eq.) was added to a solution of anhydrous AlCl_3_ (0.7 g, 5.3 mmol, 2.2 eq.) in CH_2_Cl_2_ (6 mL) and the solution was added dropwise to the cooled solution of [2.2]paracyclophane. The reaction mixture was warmed to room temperature and stirred at rt for 4 h. After completion of the transformation, saturated Na_2_CO_3_ solution (20 mL) was added to the reaction mixture. The organic layer was separated, and the aqueous layer was extracted with CH_2_Cl_2_ (3×20 mL). The organic layer was dried (Na_2_SO_4_) and concentrated *in vacuo*. The residue was purified by FCC (Ø=3 cm, l=15 cm, V=70 mL, cyclohexane/ethyl acetate 98 : 2). R_f_=0.75 (cyclohexane/ethyl acetate 9 : 1). Colorless solid, mp 205 °C, yield 0.58 g (85 %). C_18_H_17_ClO (284.8 g/mol). ^1^H NMR (400 MHz, DMSO‐D_6_): δ (ppm)=2.84 (ddd, *J*=12.5/10.1/6.5 Hz, 1H, 2‐H), 2.91–3.16 (m, 6H, C*H*_*2*(paracyclophane)_), 3.71 (ddd, *J*=12.0/9.8/1.8 Hz, 1H, 2‐H), 4.79 (d, *J*=15.8 Hz, 1H, C*H*_2_Cl), 5.03 (d, *J*=15.8 Hz, 1H, C*H*_2_Cl), 6.30 (dd, *J*=7.8/1.8 Hz, 1H, 12‐H), 6.42 (dd, *J*=7.8/1.9 Hz, 1H, 13‐H), 6.50 (dd, *J*=7.8/1.9 Hz, 1H, 15‐H), 6.54 (dd, *J*=7.8/1.9 Hz, 1H, 16‐H), 6.58 (d, *J*=7.8 Hz, 1H, 8‐H), 6.74 (dd, *J*=7.7/1.8 Hz, 1H, 7‐H), 7.11 (d, *J*=1.8 Hz, 1H, 5‐H). ^13^C NMR (101 MHz, DMSO‐D_6_): δ (ppm)=34.7 (C‐10), 34.8 (C‐1), 35.0 (C‐9), 35.5 (C‐2), 49.0 (*C*H_2_Cl), 131.4 (C‐12), 132.7 (C‐13), 133.1 (C‐15), 133.4 (C‐16), 134.2 (C‐5), 135.1 (C‐3), 136.8 (C‐8), 137.5 (C‐7), 139.6 (C‐14), 139.7 (C‐11), 140.4 (C‐6), 141.7 (C‐4), 193.3 (C=O).

### (±)‐2‐Azido‐1‐([2.2]paracyclophan‐4‐yl)ethan‐1‐one (9)


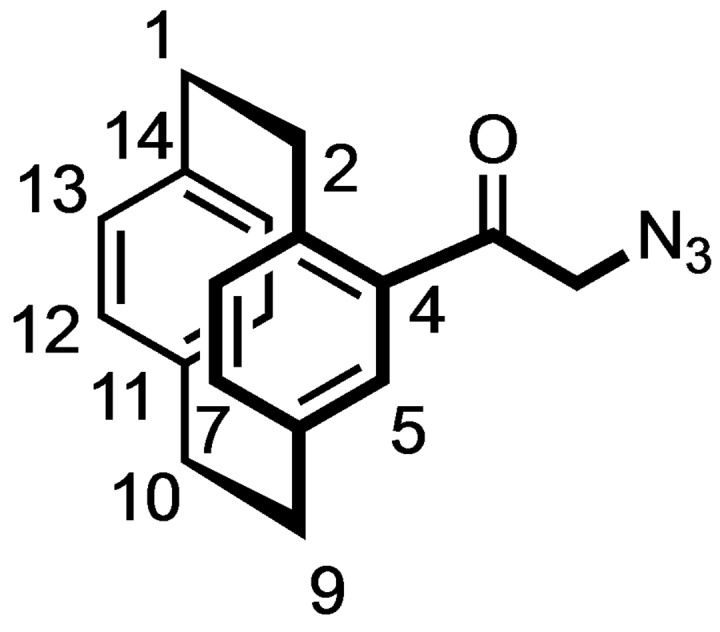


Chloroacetylparacyclophane **7** (0.50 g, 1.7 mmol, 1.0 eq.) was dissolved in DMF (10 mL) and a catalytic amount of KI was added. The reaction mixture was stirred for 10 min. Then, NaN_3_ (0.20 g, 3.16 mmol, 1.8 eq.) was added and the resulting mixture was stirred at rt for 10 h. Et_2_O (30 mL) was added to the reaction mixture followed by addition of H_2_O (30 mL). The organic layer was separated, and the aqueous layer was extracted with CH_2_Cl_2_ (3×30 mL). The organic layer was dried (Na_2_SO_4_) and concentrated in vacuo. The residue was purified by FCC (Ø=3 cm, l=20 cm, V=75 mL, cyclohexane/ethyl acetate 98 : 2). R_f_=0.73 (cyclohexane/ethyl acetate 9 : 1). Yellow oil, yield 0.51 g (99 %). C_18_H_17_N_3_O (291.4 g/mol). ^1^H NMR (400 MHz, CD_3_OD): δ (ppm)=2.88 (ddd, *J*=12.6/9.9/6.9 Hz, 1H, 2‐H), 2.97–3.22 (m, 6H, C*H*_*2*(paracyclophane)_), 3.89 (ddd, *J*=12.1/9.0/2.6 Hz, 1H, 2‐H), 4.24 (d, *J*=17.8 Hz, 1H, C*H*_2_N_3_), 4.62 (d, *J*=17.8 Hz, 1H, C*H*_2_N_3_), 6.40 (s, 2H, 12‐H, 13‐H), 6.51 (d, *J*=7.6 Hz, 1H, 15‐H), 6.56 (d, *J*=7.6 Hz, 1H, 16‐H), 6.60 (d, *J*=7.8 Hz, 1H, 8‐H), 6.75 (dd, *J*=7.8/1.8 Hz, 1H, 7‐H), 6.96 (d, *J*=1.8 Hz, 1H, 5‐H). ^13^C NMR (151 MHz, CD_3_OD): δ (ppm)=37.0 (C‐10), 37.1 (C‐1), 37.2 (C‐9), 38.0 (C‐2), 58.2 (*C*H_2_N_3_), 133.4 (C‐12), 134.5 (C‐13), 135.3 (C‐15), 135.6 (C‐16), 135.7 (C‐5), 137.5 (C‐3), 139.1 (C‐8), 139.8 (C‐7), 142.0 (C‐14), 142.4 (C‐11), 143.0 (C‐6), 144.5 (C‐4), 198.8 (C=O).

### (±)‐1‐([2.2]Paracyclophan‐4‐yl)‐2‐(4‐phenyl‐1H‐1,2,3‐triazol‐1‐yl)ethan‐1‐one (10)


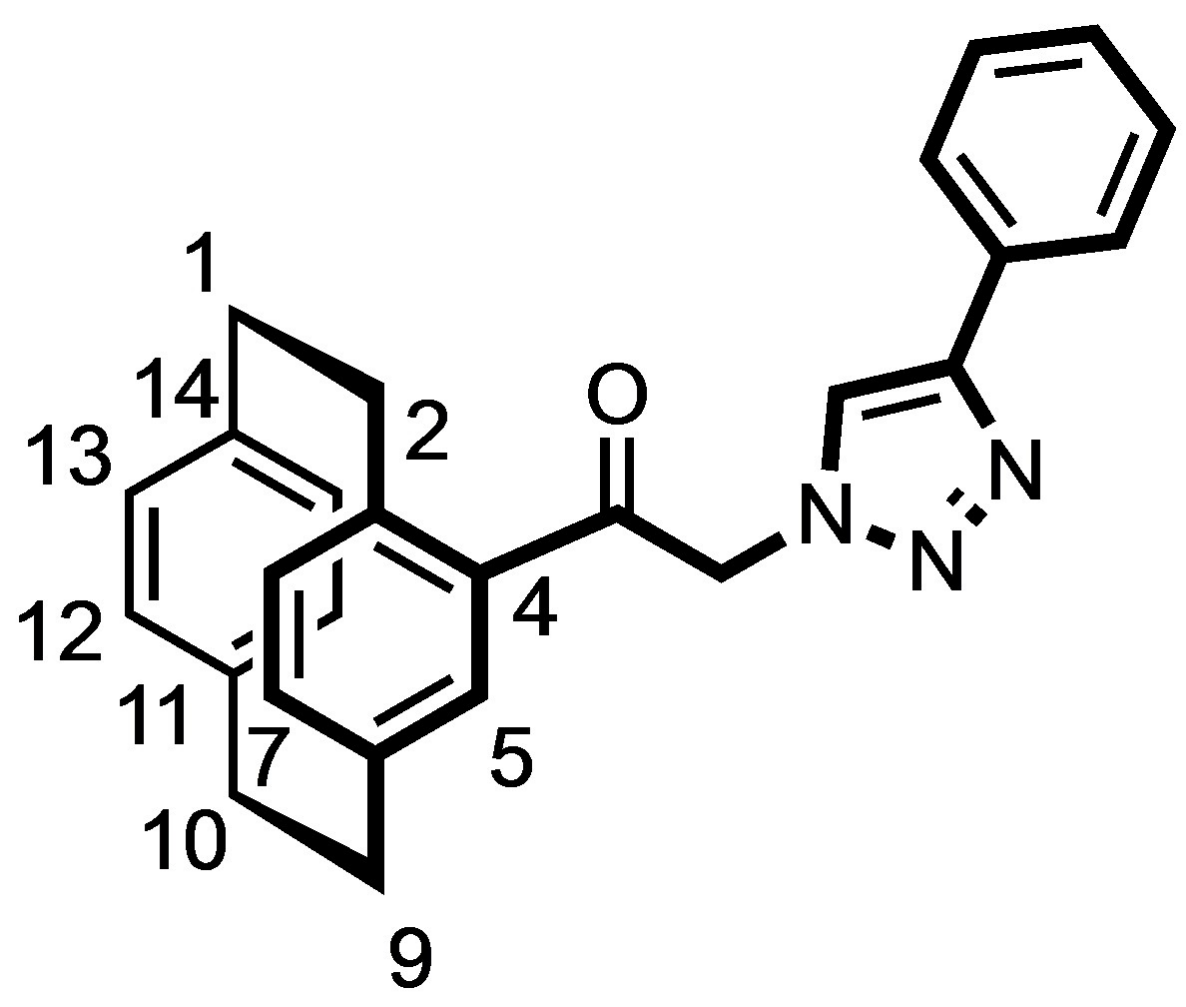


A solution of CuSO_4_.5H_2_O (3.8 mg, 0.0015 mmol, 0.01 equiv.) and sodium ascorbate (6.1 mg, 0.003 mmol, 0.02 equiv.) in H_2_O (2 mL) was added to a mixture of phenylacetylene (17.1 μL, 0.15 mmol, 1.0 equiv.) and the azide **9** (50 mg, 0.17 mmol, 1.1 equiv.) at rt. The resulting mixture was stirred until the reaction mixture was solidified completely (10 min). The solid residue was dissolved in CH_2_Cl_2_ (20 mL) and H_2_O (20 mL) was added. The organic layer was separated, and the aqueous layer was extracted with CH_2_Cl_2_ (3×20 mL). The organic layer was dried (Na_2_SO_4_) and concentrated in vacuo. The residue was purified by FCC (Ø=2 cm, l=15 cm, V=30 mL, cyclohexane/ethyl acetate 4 : 1). R_f_=0.51 (cyclohexane/ethyl acetate 2 : 1). Colorless solid, mp 178 °C, yield 43 mg (86 %). C_26_H_23_N_3_O (393.5 g/mol). ^1^H NMR (300 MHz, CDCl_3_): δ (ppm)=2.75–2.91 (m, 1H, 2‐H), 2.91–3.26 (m, 6H, C*H*_*2*(paracyclophane)_), 3.80 (ddd, *J*=11.9/9.1/2.6 Hz, 1H, 2‐H), 5.25 (d, *J*=17.4 Hz, 1H, COC*H*_2_), 5.83 (d, *J*=17.4 Hz, 1H, COC*H*_2_), 6.29–6.40 (m, 2H, 12‐H, 13‐H), 6.43–6.53 (m, 2H, 15‐H, 16‐H), 6.55 (d, *J*=7.8 Hz, 1H, 8‐H), 6.71 (dd, *J*=7.8/1.9 Hz, 1H, 7‐H), 6.98 (d, *J*=1.7 Hz, 1H, 5‐H), 7.29 (t, *J*=7.7 Hz, 1H, 4‐H_phenyl_), 7.39 (t, *J*=7.8 Hz, 2H, 3‐H_phenyl_, 5‐H_phenyl_), 7.84 (d, *J*=7.3 Hz, 2H, 2‐H_phenyl_, 6‐H_phenyl_), 7.90 (s, 1H, H_triaz_). ^13^C NMR (151 MHz, CD_3_Cl_3_): δ (ppm)=37.5 (C‐10), 37.8 (C‐1), 37.8 (C‐9), 38.6 (C‐2), 59.0 (C(=O)*C*H_2_triaz), 124.1 (C‐5_triaz_), 128.5 (2 C, C‐2_phenyl_, C‐6_phenyl_), 130.9 (C‐4_phenyl_), 131.5 (2 C, C‐3_phenyl_, C‐5_phenyl_), 133.1 (C‐1_phenyl_), 133.8 (C‐12), 134.8 (C‐13), 135.6 (C‐5), 135.6 (C‐15), 135.7 (C‐16), 137.0 (C‐3), 139.5 (C‐8), 140.5 (C‐7), 141.9 (C‐14), 142.7 (C‐11), 143.2 (C‐6), 145.5 (C‐4), 150.8 (C‐4_triaz_), 194.6 (C=O).

### (±)‐2‐Amino‐1‐([2.2]paracyclophan‐4‐yl)ethan‐1‐one (11)


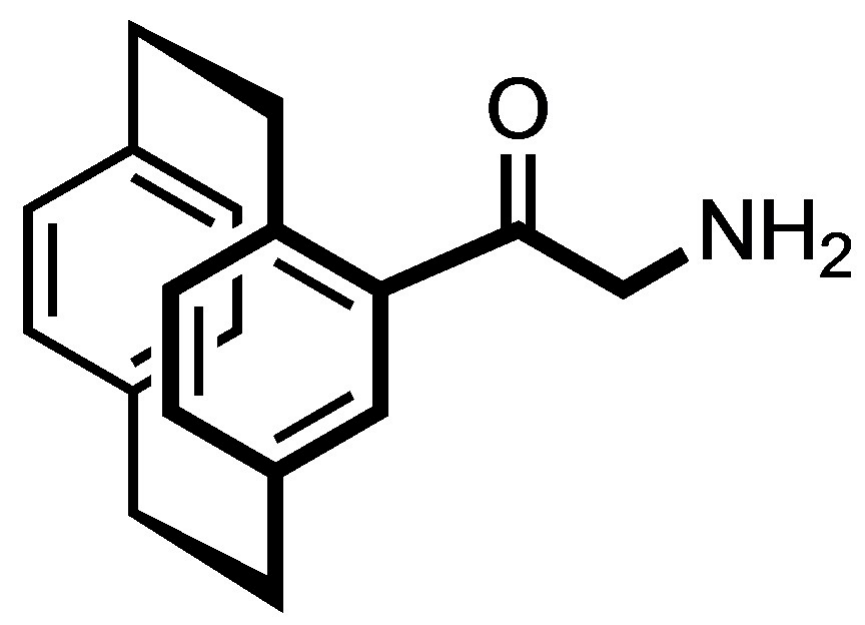


Azidoacetylparacyclophane **9** (0.5 g, 1.7 mmol, 1.0 eq.) was dissolved in a mixture of THF: MeOH (1 : 1, 10 mL) and 5 % Pd/C (50 mg) was added. The mixture was flushed thrice with H_2_ gas. The flask was filled with H_2_ (5 bar) and the mixture was stirred at rt for 16 h. Pd/C was removed by passing the reaction mixture through a Celite® 545 bed. The filtrate was concentrated in vacuo. The residue was obtained as a yellow oil, which was proceeded to the next step without further purification. Yellow oil, yield 0.39 g (86 %). R_f_=0.51 (CH_2_Cl_2_: CH_3_OH 9 : 1). C_18_H_19_NO (265.4 g/mol).

### (±)‐N‐[2‐Oxo‐2‐([2.2]paracyclophan‐4‐yl)ethylbenzamide (12 a)


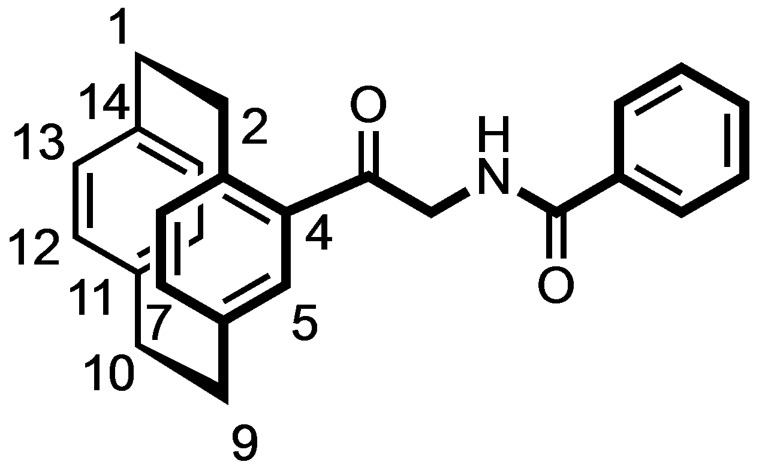


Benzoic acid (46 mg, 0.4 mmol, 1.0 equiv.), amine **11** (100 mg, 0.4 mmol, 1.0 equiv.) and COMU (177 mg, 0.44 mmol, 1.1 equiv.) were suspended in THF (15 mL). After addition of DIPEA (133 μL, 0.8 mmol, 2.0 equiv.), the resulting yellow solution was stirred at rt overnight. The solvent was removed under reduced pressure and water (20 mL) was added to the residue. The resulting yellow solution was stirred for 30 min at rt and ethyl acetate (20 mL) was added. The organic phase was separated, and the aqueous layer was extracted with ethyl acetate (3×20 mL). The organic layer was dried (Na_2_SO_4_) and concentrated in vacuo. The residue was purified by FCC (Ø=2 cm, l=15 cm, V=15 mL, cyclohexane:ethyl acetate 9 : 1). R_f_=0.46 (cyclohexane:ethyl acetate 4 : 1). Light yellow oil, yield 51 mg (88 %). C_25_H_23_NO_2_ (369.5 g/mol). ^1^H NMR (600 MHz, DMSO‐D_6_): δ (ppm)=2.83 (ddd, *J*=12.5/10.1/6.8 Hz, 1H, 2‐H), 3.01–3.17 (m, 6H, C*H*_*2*(paracyclophane)_), 3.81–3.89 (m, 1H, 2‐H), 4.36 (dd, *J*=17.6/5.9 Hz, 1H, COC*H*_2_NH), 4.71 (dd, *J*=17.6/5.8 Hz, 1H, COC*H*_2_NH), 6.48–6.51 (m, 2H, 12‐H, 13‐H), 6.53–6.57 (m, 2H, 15‐H, 16‐H), 6.59 (d, *J*=7.7 Hz, 1H, 8‐H), 6.73 (dd, *J*=7.8/1.8 Hz, 1H, 7‐H), 7.21 (d, *J*=1.8 Hz, 1H, 5‐H), 7.50 (t, *J*=7.7 Hz, 2H, 3‐H_phenyl_, 5‐H_phenyl_), 7.54–7.57 (m, 1H, 4‐H_phenyl_), 7.94 (dd, *J*=8.7/2.0 Hz, 2H, 2‐H_phenyl_, 6‐H_phenyl_), 8.80 (t, *J*=5.7 Hz, 1H, CH_2_N*H*CO). ^13^C NMR (151 MHz, DMSO‐D_6_): δ (ppm)=37.4 (C‐10), 37.5 (C‐1), 37.7 (C‐9), 38.2 (C‐2), 50.6 (CO*C*H_2_NH), 130.4 (2 C, C‐2_phenyl_, C‐6_phenyl_), 131.5 (2 C, C‐3_phenyl_, C‐5_phenyl_), 132.4 (C‐4_phenyl_), 134.4 (C‐12), 134.5 (C‐3), 135.1 (C‐13), 136.2 (C‐15), 136.7 (C‐5), 137.2 (C‐16), 138.8 (C‐1_phenyl_), 139.4 (C‐8), 139.7 (C‐7), 142.3 (C‐14), 142.6 (C‐11), 143.0 (C‐6), 144.1 (C‐4), 169.7 (*C*(=O)NH), 199.5 (C=O).

**(±)‐*N*‐[2‐Oxo‐2‐([2.2]paracyclophan‐4‐yl)ethyl‐2‐iodobenzamide (12 b)**


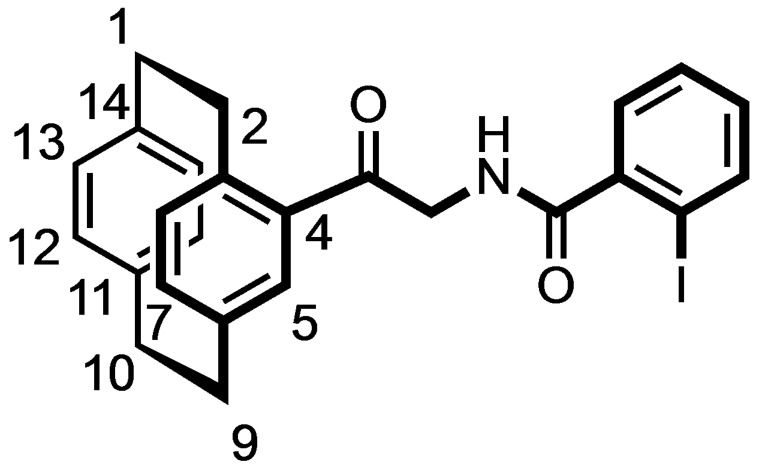


2‐Iodobenzoic acid (18 mg, 0.07 mmol, 1.0 equiv.), amine **11** (20 mg, 0.07 mmol, 1.0 equiv.) and COMU (36 mg, 0.077 mmol, 1.1 equiv.) were suspended in THF (10 mL). After addition of DIPEA (26 μL, 0.14 mmol, 2.0 equiv.), the resulting yellow solution was stirred at rt overnight. The solvent was removed under reduced pressure and H_2_O (10 mL) was added to the residue. The resulting yellow solution was stirred for 30 min at rt and ethyl acetate (20 mL) was added. The organic phase was separated, and the aqueous layer was extracted with ethyl acetate (3×20 mL). The organic layer was dried (Na_2_SO_4_) and concentrated in vacuo. The residue was purified by FCC (Ø=2 cm, l=10 cm, V=15 mL, cyclohexane:ethyl acetate 9 : 1). R_f_=0.5 (cyclohexane:ethyl acetate 4 : 1). Yellow oil, yield 51 mg (84 %). C_25_H_22_INO_2_ (495.4 g/mol). ^1^H NMR (600 MHz, DMSO‐D_6_): δ (ppm)=2.83 (ddd, *J*=12.5/10.0/6.8 Hz, 1H, 2‐H), 3.01–3.17 (m, 6H, C*H*_*2*(paracyclophane)_), 3.83 (ddd, *J*=12.2/9.9/1.7 Hz, 1H, 2‐H), 4.39 (dd, *J*=17.7/5.7 Hz, 1H, COC*H*_2_NH), 4.76 (dd, *J*=17.7/5.7 Hz, 1H, COC*H*_2_NH), 6.48–6.51 (m, 2H, 2H, 12‐H, 13‐H), 6.53–6.56 (m, 2H, 15‐H, 16‐H), 6.60 (d, *J*=7.7 Hz, 1H, 8‐H), 6.74 (dd, *J*=7.7/1.8 Hz, 1H, 7‐H), 7.17–7.21 (m, 2H, 5‐H_phenyl_, 5‐H), 7.44–7.51 (m, 2H, 4‐H_phenyl_, 6‐H_phenyl_), 7.91 (dd, *J*=7.9/1.0 Hz, 1H, 3‐H_phenyl_), 8.67 (t, *J*=5.8 Hz, 1H, CH_2_N*H*CO). ^13^C NMR (151 MHz, DMSO‐D_6_): δ (ppm)=37.4 (C‐10), 37.5 (C‐1), 37.7 (C‐9), 38.2 (C‐2), 50.6 (CO*C*H_2_NH), 131.1 (C‐4_phenyl_), 131.6 (C‐6_phenyl_), 134.1 (C‐5_phenyl_), 134.4 (C‐12), 135.2 (C‐13), 135.9 (C‐15), 136.2 (C‐16), 136.7 (C‐5), 138.8 (C‐3), 139.4 (C‐8), 139.7 (C‐7), 141.9 (C‐1_phenyl_), 142.3 (C‐14), 142.4 (C‐3_phenyl_), 142.6 (C‐11), 143.0 (C‐6), 144.1 (C‐4), 145.4 (C‐2_phenyl_), 172.3 (*C*(=O)NH), 199.3 (C=O).

### Pharmacological evaluation

#### Molecular biology and electrophysiology (TEVC)

Molecular biology and TEVC experiments were conducted as previously described.[^25^, ^26^, ^27^] Measurements were performed using a Turbo Tec 10CX amplifier (NPI electronic, Tamm, Germany), NI USB 6221 DA/AD Interface (National Instruments, Austin, USA) and GePulse Software (Dr. Michael Pusch, Genova, Italy). All experiments were conducted at room temperature. The agonist solutions were freshly prepared on the day of measurement from 100 mM stock solutions of glycine and glutamate and final concentrations of 10 μM each of the agonists were obtained. The test compound solutions were prepared from 10 mM DMSO stocks by diluting with agonist solutions and final concentrations of 10 μM for each test compound were obtained. The oocytes were superfused with Ba^2+^‐Ringer containing (mmol/L): 10 HEPES, 90 NaCl, 1 KCl, 1.5 BaCl_2_ during measurements. The pH of the Ba^2+^‐Ringer solution was adjusted to 7.4 with 1 M NaOH. The recording pipettes were filled with 3 M KCl. The currents were measured at a holding potential of −70 mV.

The data from the measurements were analyzed using Ana (Dr. Michael Pusch, Genova, Italy), Origin and GraphPad Prism 3. The inhibition of test compounds was calculated by the equation:

$$Inhibition=1-\frac{Ic-Ib}{Ia-Ib}$$

Where represents the Ic represents the resting current in presence of the test compound solution, Ib represents the holding current before the agonist addition and Ia represents the current after agonist addition. For comparing the inhibitory activity of the test compounds, the inhibition of each test compound was normalized to the inhibition by the lead compound **1**. The normalized inhibition was calculated by the following equation:

$$Inorm\;\left(\%\right)=\;\frac{Inhibition\;of\;compound\;at\;10\;\;\mu M}{Inhibition\;of\;TCN-201\;at\;10\;\;\mu M}\;x\;100$$

The significance of Inorm was tested by One‐way‐ANOVA and post hoc mean comparison Tukey Test.

#### Receptor binding studies

The affinity towards the ifenprodil binding site of GluN2B subunit‐containing NMDA receptor, σ_1_ und σ_2_ receptors were measured using radioligand receptor binding assay. The affinity towards the GluN2B subunit‐containing NMDA receptor was recorded as given in the literature.[^31^, ^32^] Similarly the experimental procedures for the radioligand receptor binding assay for the σ_1_ und σ_2_ receptors are also reported.[^33^, ^34^, ^35^] Details are given in the Supporting Information.

## Supporting Information

The Supporting Information contains MS data of all synthesized compounds as well as purity data of all test compounds. Furthermore, details of the receptor binding studies, affinity data towards σ_1_, σ_2_ and GluN2B receptors as well as One‐Way‐ANOVA data analysis are given. The ^1^H and ^13^C NMR spectra of all prepared compounds are shown.

## Abbreviations

AMPA: α‐amino‐3‐hydroxy‐5‐methyl‐4‐isoxazolepropionic acid
COMU: (1‐cyano‐1‐ethoxycarbonylmethylenaminoxy)‐dimethylaminomorpholinocarbenium hexafluorophosphate
DIPEA: *N*,*N*‐diisopropylethylamine
iGluR: ionotropic glutamate receptor
LTD: long‐term depression
LTP: long‐term potentiation
NAM: negative allosteric modulator
THF: tetrahydrofuran

## Conflict of interest

The authors declare no conflicts of interest.

## Supporting information

As a service to our authors and readers, this journal provides supporting information supplied by the authors. Such materials are peer reviewed and may be re‐organized for online delivery, but are not copy‐edited or typeset. Technical support issues arising from supporting information (other than missing files) should be addressed to the authors.

Supporting Information
