# Supplementary material for: [2.2]Paracyclophane‐Based TCN‐201 Analogs as GluN2A‐Selective NMDA Receptor Antagonists
Source: ChemMedChem. 2021 Aug 3;16(20):3201–9. doi: 10.1002/cmdc.202100400 (PMC8596836; doi:10.1002/cmdc.202100400)
Supplement: Supplementary file 1 — Supporting Information [file CMDC-16-3201-s001.pdf]

ChemMedChem

Supporting Information

[2.2]Paracyclophane-Based TCN-201 Analogs as GluN2A-Selective NMDA Receptor Antagonists

Remya Rajan, Dirk Schepmann, Ruben Steigerwald, Julian A. Schreiber, Ehab El-Awaad, Joachim Jose, Guiscard Seebohm, and Bernhard Wünsch*

Table of Contents

1.	Mass spectrometric data of the test compounds	S2
2.	Purity of test compounds	S3
3.	Receptor binding studies	S4
4.	One-way-ANOVA	S9
5.	References	S10
6.	^{1}H and ^{13}C NMR spectra of the synthesized compounds	S11

1. Mass spectrometric data of the test compounds

Table SI1: Exact mass of all synthesized compounds.

compound	exact mass found	exact mass calculated
**7**	285.1027	285.1041 ($[M+H]^+$)
**8**	356.1918	356.2014 [M-CO]
**9**	292.1456	292.1444 ($[M+H]^+$)
**10**	394.1930	394.1914 ($[M+H]^+$)
**11**	266.1569	266.1539 ($[M+H]^+$)
**12a**	370.1820	370.1802 ($[M+H]^+$)
**12b**	496.0792	496.768 ($[M+H]^+$)

2. Purity of test compounds

Table SI2: HPLC analysis of the test compounds.

compound	retention time (t_R) (min)	purity (%)
**8**	25.2	98.7
**10**	15.1	98.1
**12a**	15.0	98.7
**12b**	15.1	99.6

3. Receptor binding studies

3.1. Materials

Guinea pig brains, rat brains and rat livers were commercially available from Harlan-Winkelmann, Borchen, Germany. Pig brains were a donation of the local slaughterhouse (Coesfeld, Germany). The mouse recombinant L(tk) cells stably expressing the GluN2B subunit-containing NMDAR were obtained from Prof. Dr. Dieter Steinhilber (Frankfurt, Germany). Homogenizers: Elvehjem Potter (B. Braun Biotech International, Melsungen, Germany) and Soniprep® 150, MSE, London, UK). Centrifuges: Cooling centrifuge model Rotina® 35R (Hettich, Tuttlingen, Germany) and High-speed cooling centrifuge model Sorvall® RC-5C plus (Thermo Fisher Scientific, Langenselbold, Germany). Multiplates: standard 96-well multiplates (Diagonal, Münster, Germany). Shaker: self-made device with adjustable temperature and tumbling speed (scientific workshop of the institute). Harvester: MicroBeta® FilterMate 96 Harvester. Filter: Printed Filtermat Typ A and B. Scintillator: Meltilex® (Typ A or B) solid state scintillator. Scintillation analyzer: MicroBeta® Trilux (all Perkin Elmer LAS, Rodgau-Jügesheim, Germany).

3.2. Preparation of membrane homogenates from guinea pig brain

Five guinea pig brains were homogenized with the potter (500-800 rpm, 10 up and down strokes) in 6 volumes of cold 0.32 M sucrose. The suspension was centrifuged at 1,200 x g for 10 min at 4 °C. The supernatant was separated and centrifuged at 23,500 x g for 20 min at 4 °C. The pellet was resuspended in 5-6 volumes of buffer (50 mM TRIS, pH 7.4) and centrifuged again at 23,500 x g (20 min, 4 °C). This procedure was repeated twice. The final pellet was resuspended in 5-6 volumes of buffer and frozen (-80 °C) in 1.5 mL portions containing about 1.5 mg protein/mL.

3.3. Preparation of membrane homogenates from rat liver

Two rat livers were cut into small pieces and homogenized with the potter (500-800 rpm, 10 up and down strokes) in 6 volumes of cold 0.32 M sucrose. The suspension was centrifuged at 1,200 x g for 10 min at 4 °C. The supernatant was separated and centrifuged at 31,000 x g for 20 min at 4 °C. The pellet was resuspended in 5-6 volumes of buffer (50 mM TRIS, pH 8.0) and incubated at rt for 30 min. After the incubation, the

suspension was centrifuged again at 31,000 x g for 20 min at 4 °C. The final pellet was resuspended in 5-6 volumes of buffer and stored at -80 °C in 1.5 mL portions containing about 2 mg protein/mL.

3.4. Preparation of cells for GluN2B binding assay from mouse liver

Mouse L(tk) cells stably transfected with the dexamethasone-inducible eukaryotic expression vectors pMSG GluN1a, pMSG GluN2B (1:5 ratio) were grown in Modified Earl's Medium (MEM) containing 10 % of standardized FCS (Biochrom AG, Berlin, Germany). The expression of the NMDA receptor at the cell surface was induced after the cell density of the adherent growing cells had reached approximately 90 % of confluency. For the induction, the original growth medium was replaced by growth medium containing 4 µM dexamethasone and 4 µM ketamine (final concentration). After 24 h, the cells were rinsed with phosphate buffered saline solution (PBS, Biochrom AG, Berlin, Germany), harvested by mechanical detachment and pelleted (10 min, 5,000 x g). For the binding assay, the cell pellet was resuspended in PBS solution and the number of cells was determined using a Scepter® cell counter (MERCK Millipore, Darmstadt, Germany). Subsequently, the cells were lysed by sonication (4 °C, 6 x 10 s cycles with breaks of 10 s). The resulting cell fragments were centrifuged with a high-performance cool centrifuge (23,500 x g, 4 °C). The supernatant was discarded, and the pellet was resuspended in a defined volume of PBS yielding cell fragments of approximately 500,000 cells/mL. The suspension of membrane homogenates was sonicated again (4 °C, 2 x 10 s cycles with a break of 10 s) and stored at -80 °C.

3.5. Determination of Protein concentration

The protein concentration was determined by the method of Bradford,[1] modified by Stoscheck.[2] The Bradford solution was prepared by dissolving 5 mg of Coomassie Brilliant Blue G 250 in 2.5 mL of EtOH (95 %, v/v). 10 mL deionized H_2O and 5 mL phosphoric acid (85 %, m/v) were added to this solution, the mixture was stirred and filled to a total volume of 50 mL with deionized water. The calibration was carried out using bovine serum albumin as a standard in 9 concentrations (0.1, 0.2, 0.4, 0.6, 0.8, 1.0, 1.5, 2.0 and 4.0 mg /mL). In a 96 well standard multiplate, 10 µL of the calibration solution or

10 µL of the membrane receptor preparation were mixed with 190 µL of the Bradford solution, respectively. After 5 min, the UV absorption of the protein-dye complex at λ = 595 nm was measured with a plate reader (Tecan Genios®, Tecan, Crailsheim, Germany).

3.6. General procedures for the binding assays

The test compound solutions were prepared from the 10 mM stock solution. To obtain the required test solutions for the assay, the DMSO stock solution was diluted with the respective assay buffer. The filtermats were presoaked in 0.5 % aqueous polyethyleneimine solution for 2 h at room temperature before use. All binding experiments were carried out in duplicates in the 96-well multiplates. Generally, the assays were performed by addition of 50 µL of the respective assay buffer, 50 µL of test compound solution in various concentrations (10^{-5}, 10^{-6}, 10^{-7}, 10^{-8}, 10^{-9} and 10^{-10} mol/L), 50 µL of the corresponding radioligand solution and 50 µL of the respective receptor preparation into each well of the multiplate (total volume 200 µL). The receptor preparation was always added last. During the incubation, the multiplates were shaken at a speed of 500-600 rpm at the specified temperature. Unless otherwise noted, the assays were terminated after 120 min by rapid filtration using the harvester. During the filtration, each well was washed five times with 300 µL of water. Subsequently, the filtermats were dried at 95 °C. The solid scintillator was melted on the dried filtermats at a temperature of 95 °C for 5 min. After solidifying of the scintillator at room temperature, the trapped radioactivity in the filtermats was measured with the scintillation analyzer. Each position on the filtermat corresponding to one well of the multiplate was measured for 5 min with the [^{3}H]-counting protocol. The overall counting efficiency was 20 %.[3] The IC_{50} values were calculated with the program GraphPad Prism® 3.0 (GraphPad Software, San Diego, CA, USA) by non-linear regression analysis. Subsequently, the IC_{50} values were transformed into K_i values using the equation of Cheng and Prusoff.[3] The K_i values are given as mean value ± SEM from three independent experiments.

3.7. σ_1 Receptor affinity

The assay was performed with the radioligand [^{3}H]-(+)-pentazocine (22.0 Ci/mmol; Perkin Elmer). The thawed membrane preparation of guinea pig brain cortex (about 100 µg of the protein) was incubated with various concentrations of test compounds, 2 nM [^{3}H]-(+)-pentazocine, and TRIS buffer (50 mM, pH 7.4) at 37 °C. The non-specific binding was determined with 10 µM unlabeled (+)-pentazocine. The K_d value of (+)-pentazocine is 2.9 nM.[4]

3.8. σ_2 Receptor affinity

The assays were performed with the radioligand [^{3}H]di-*o*-tolylguanidine (specific activity 50 Ci/mmol; ARC, St. Louis, MO, USA). The thawed rat liver membrane preparation (about 100 µg protein) was incubated with various concentrations of the test compound, 3 nM [^{3}H]di-*o*-tolylguanidine and buffer containing (+)-pentazocine (500 nM (+)-pentazocine in TRIS buffer (50 mM TRIS, pH 8.0)) at rt. The non-specific binding was determined with 10 µM non-labeled di-*o*-tolylguanidine. The K_d value of di-*o*-tolylguanidine is 17.9 nM.[5]

3.9. GluN2B binding site of the NMDA receptor

The competitive binding assay was performed with the radioligand [^{3}H]-ifenprodil (60 Ci/mmol; BIOTREND, Cologne, Germany). The thawed cell membrane preparation from the transfected L(tk-) cells (about 20 µg protein) was incubated with various concentrations of test compounds, 5 nM [^{3}H]-ifenprodil, and TRIS/EDTA-buffer (5 mM TRIS/1 mM EDTA, pH 7.5) at 37 °C. The non-specific binding was determined with 10 µM unlabeled ifenprodil. The K_d value of ifenprodil is 7.6 nM.[6]

3.10. Affinity data of the [2.2]paracyclophanes 8,10, and 12

The affinity data (in %) at 1 µM concentration of the test compounds towards σ_1, σ_2 receptors and towards GluN2B subunit containing NMDA receptors is summarized in Table SI3.

Table SI3: Replacement of the corresponding radioligands (in %) by the synthesized compounds.

compound	σ_1 affinity	σ_2 affinity	GluN2B affinity
**8**	0 %	2 %	4 %
**10**	2 %	0%	9%
**12a**	10%	9%	0%
**12b**	0%	8%	4%

Inhibition of radioligand binding was measured at a concentration of 1 µM of the test compounds.

4. One-Way-ANOVA

The statistical significance of the normalized inhibition I_{norm} was tested by One-way-ANOVA and post hoc mean comparison Tukey test. The summary of the One-way-ANOVA test is given below:

	DF	Sum of Squares	Mean Square	F Value	Prob>F
Model	4	8920.15901	2230.03975	136.98173	2.11949E-7
Error	8	130.23867	16.27983		
Total	12	9050.39768			

Null hypothesis: The I_{norm} means of all the compounds are equal.

Alternative hypothesis: The I_{norm} means of one or more compounds are different.

At the 0.05 level, the I_{norm} means are significantly different.

The results of the Tukey mean comparison test is given in Table SI4.

Table SI4: Post hoc mean comparison Tukey Test for the normalized Inhibition I_{norm} of TCN-201 (**1**) and the test compounds.

	MeanDiff	SEM	q Value	Prob	Alpha	Sig	LCL	UCL
**8 – 1**	-94.2667	4.65902	28.61402	1.32E-07	0.05	1	-110.362	-78.1709
**10 – 1**	-95.3967	4.65902	28.95702	1.10E-07	0.05	1	-111.492	-79.3009
**10 – 8**	-1.13	3.29442	0.48508	0.99639	0.05	0	-12.5114	10.25143
**12a – 1**	-88.8333	4.65902	26.96476	2.86E-07	0.05	1	-104.929	-72.7376
**12a – 8**	5.43333	3.29442	2.33239	0.50964	0.05	0	-5.94809	16.81476
**12a – 10**	6.56333	3.29442	2.81748	0.34818	0.05	0	-4.81809	17.94476
**12b – 1**	-61.8133	4.65902	18.76302	7.03E-06	0.05	1	-77.9091	-45.7176
**12b – 8**	32.45333	3.29442	13.93141	6.65E-05	0.05	1	21.07191	43.83476
**12b – 10**	33.58333	3.29442	14.41649	5.16E-05	0.05	1	22.20191	44.96476
**12b – 12a**	27.02	3.29442	11.59901	2.52E-04	0.05	1	15.63857	38.40143

Sig equals to 1 indicates that the means are significantly different at the 0.05 level; Sig equals to 0 indicates that the means are not significantly different at the 0.05 level.

5. References

1. Bradford, M. M. A Rapid and sensitive Method for the Quantitation of Microgram Quantities of Protein Utilizing the Principle of Protein-Dye Binding. *Anal. Biochem*. **1976,** *72*, 248–254.
2. Stoscheck, C. Quantification of Protein. *Method. Enzymol*. **1990,** *182*, 50–68.
3. Cheng, Y. C.; Prusoff, W. H. Relationship between the inhibition constant (KI) and the concentration of inhibitor which causes 50 per cent inhibition (I50) of an enzymatic reaction. *Biochem. Pharmacol*. **1973,** *22*, 3099–3108.
4. DeHaven-Hudkins, D. L.; Fleissner, L. C.; Ford-Rice, F. Y. Characterization of the binding of [3H]-(+)-pentazocine to σ recognition sites in guinea pig brain. *Eur. J. Pharm-Molec. Ph*. **1992,** *227*, 371–378.
5. Mach, R. H.; Smith, C. R.; Childers, S. R. Ibogaine possesses a selective affinity for σ2 receptors. *Life Sci*. **1995,** *57*, PL57–PL62.
6. Schepmann, D.; Frehland, B.; Lehmkuhl, K.; Tewes, B.; Wünsch, B. Development of a selective competitive receptor binding assay for the determination of the affinity to NR2B containing NMDA receptors. *J. Pharmaceut. Biomed*. **2010,** *53*, 603–608.

6. ^{1}H and ^{13}C NMR spectra of the synthesized compounds

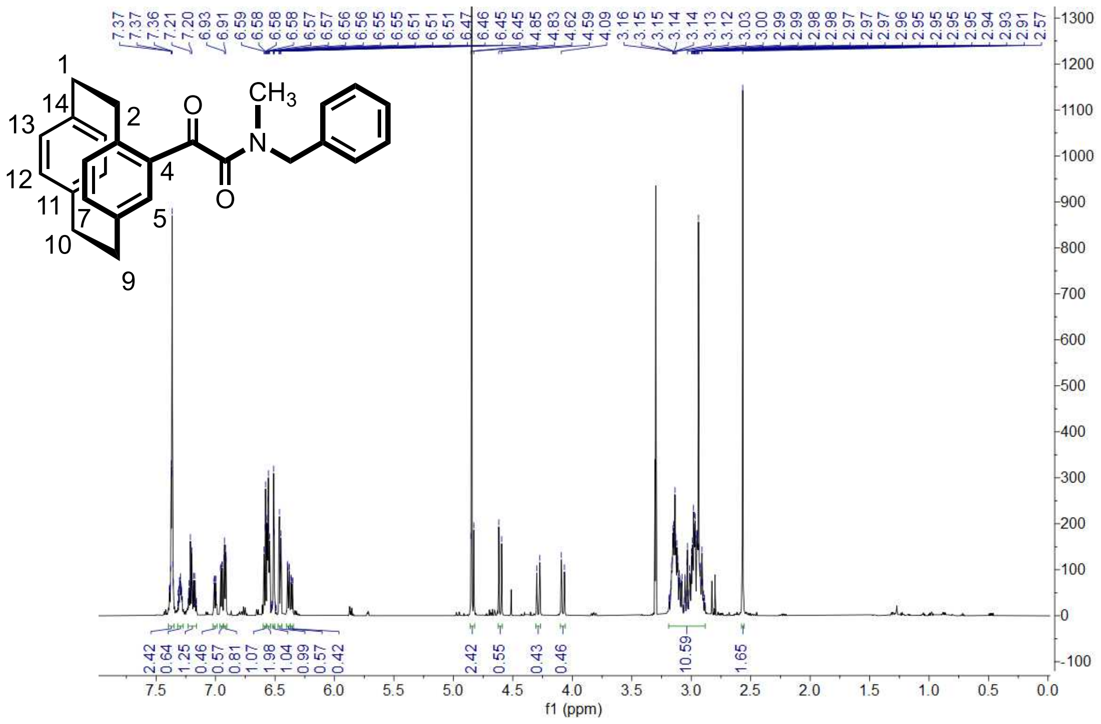

^{1}H NMR spectrum of **7** in CD_3OD

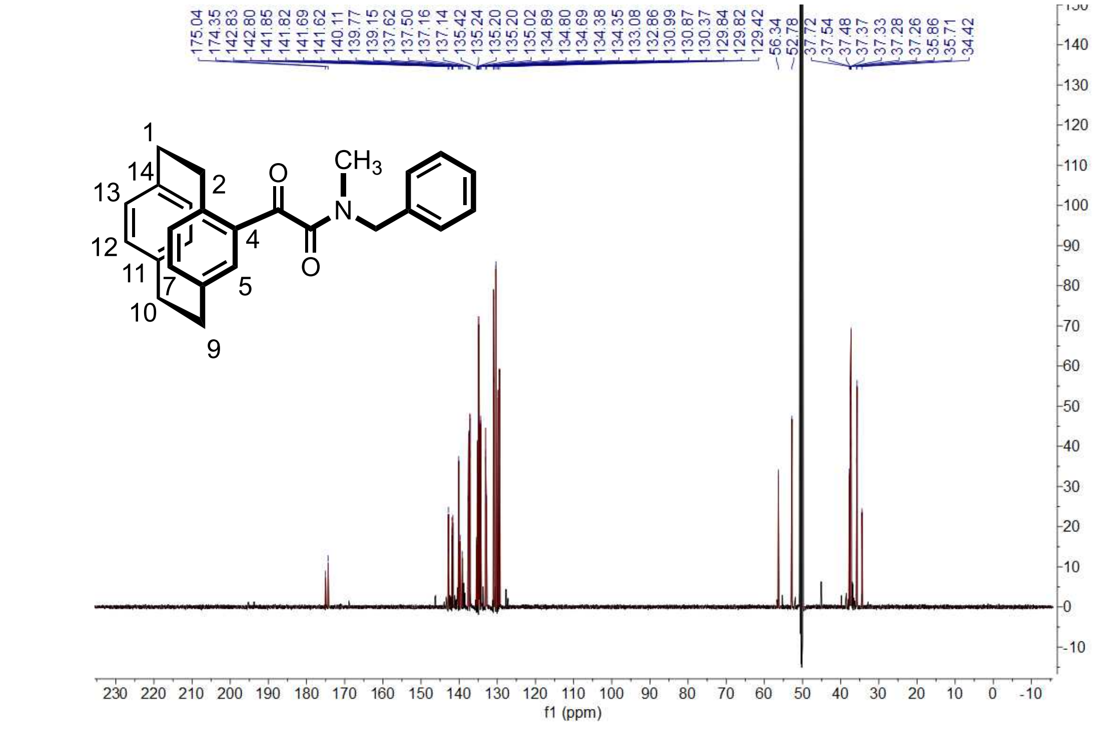

^{13}C NMR spectrum of **7** in CD_3OD

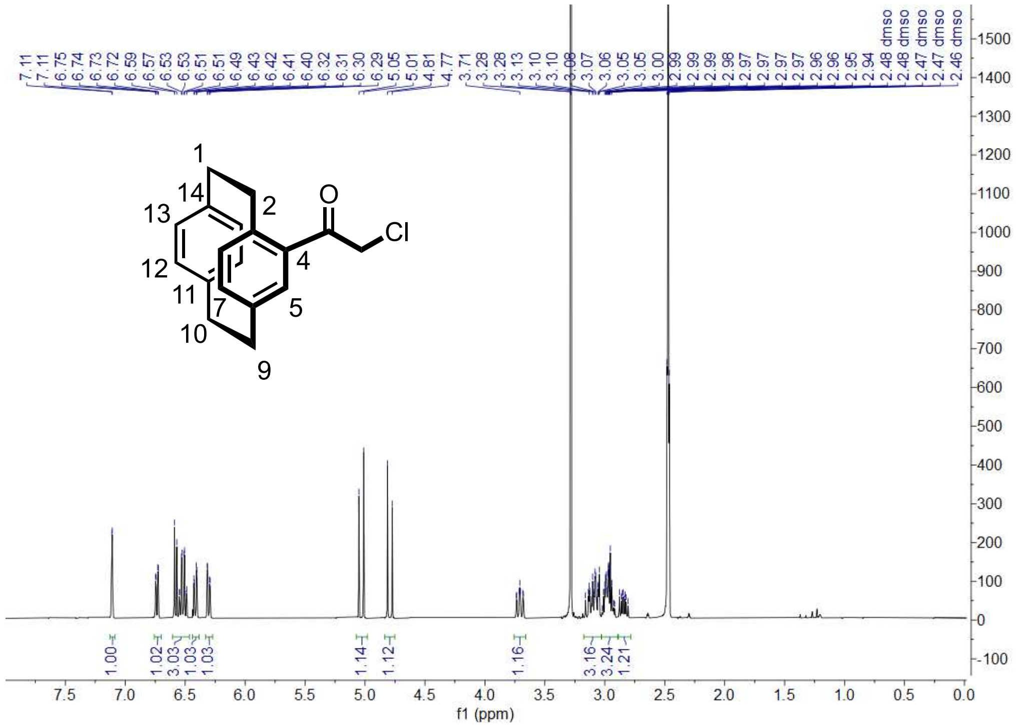

^{1}H NMR spectrum of **8** in DMSO-D_6

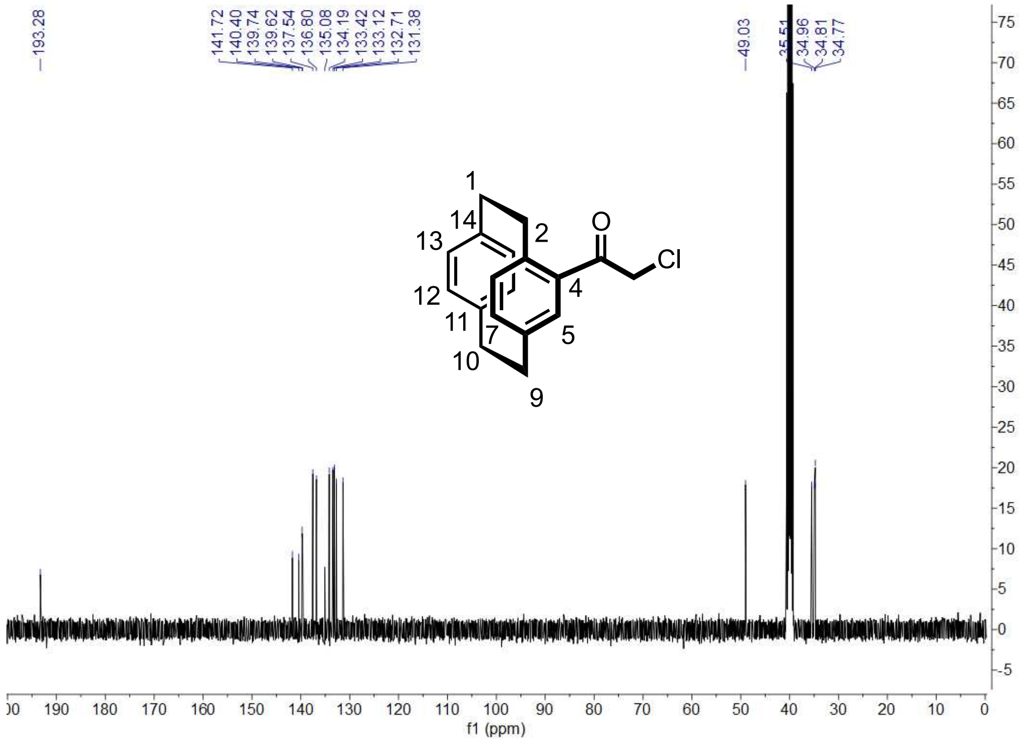

^{13}C NMR spectrum of **8** in DMSO-D_6

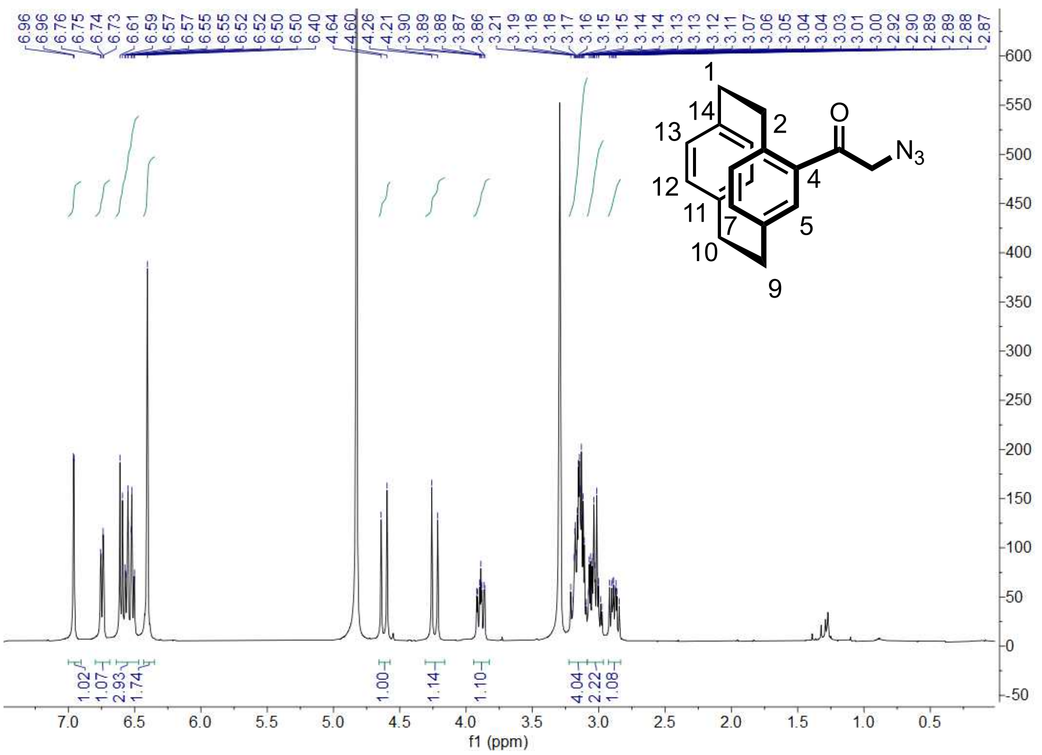

^{1}H NMR spectrum of **9** in CD_3OD

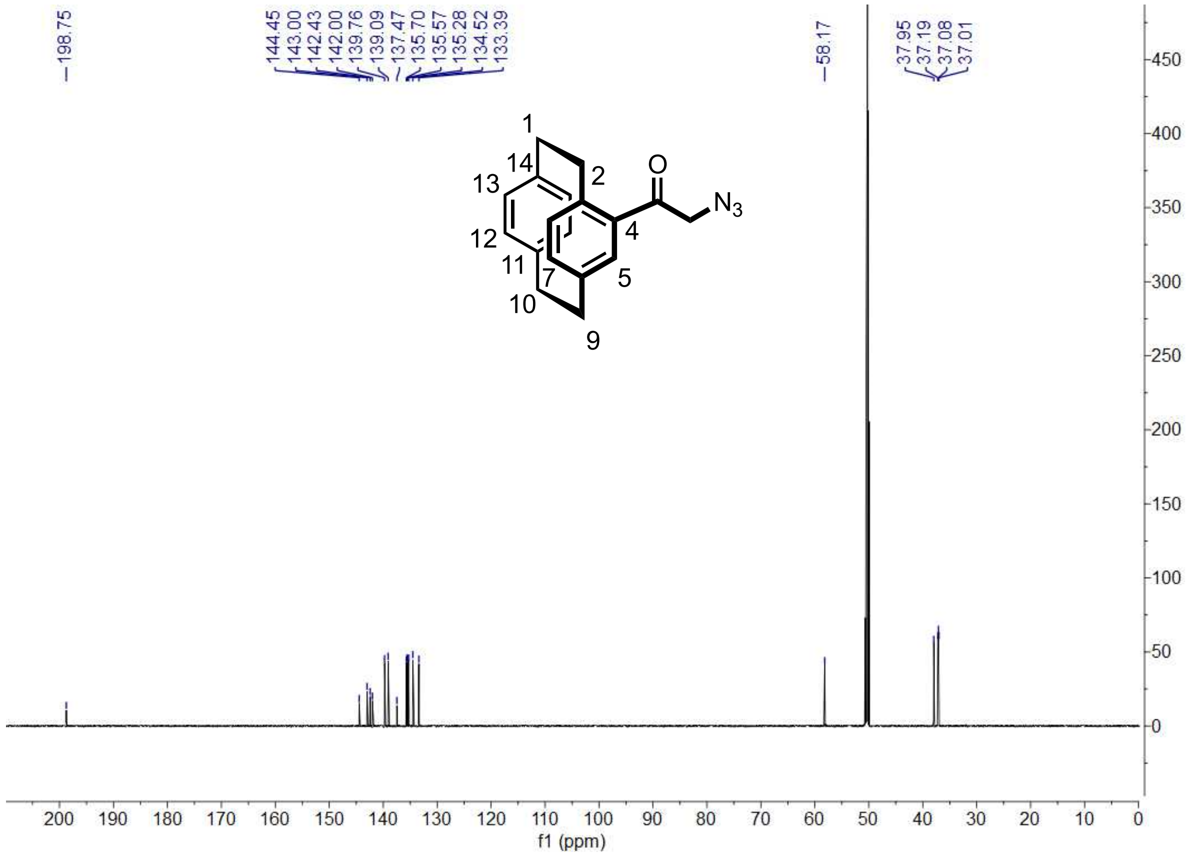

^{13}C NMR spectrum of **9** in CD_3OD

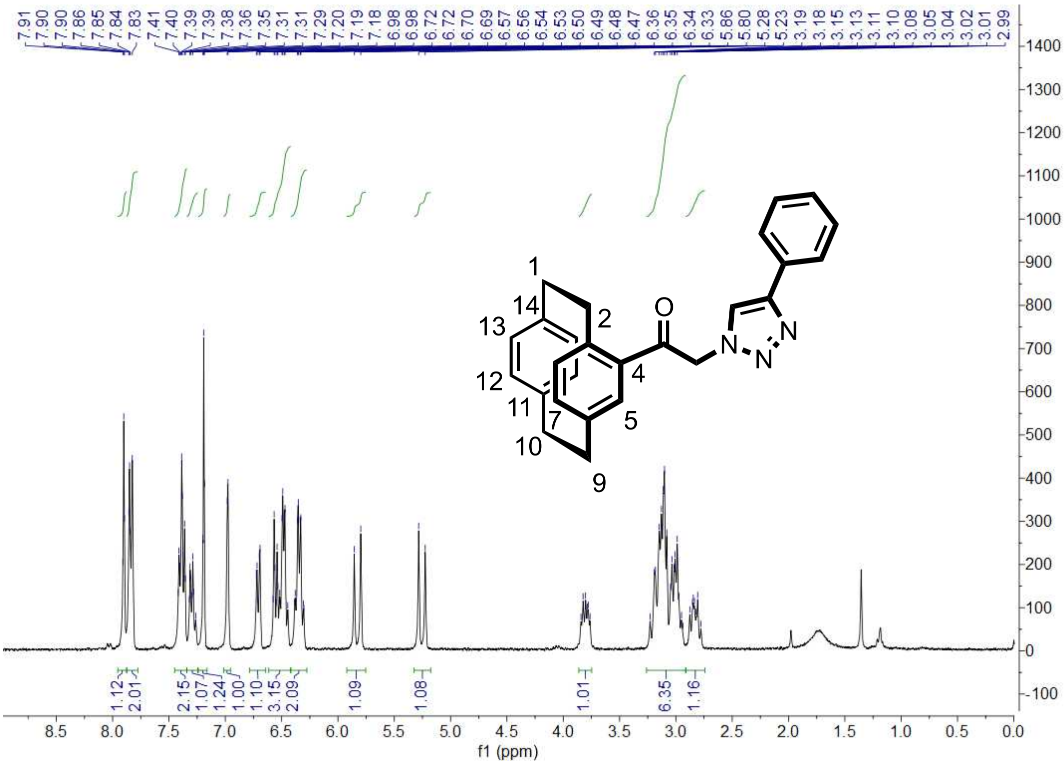

^{1}H NMR spectrum of **10** in $CDCl_3$

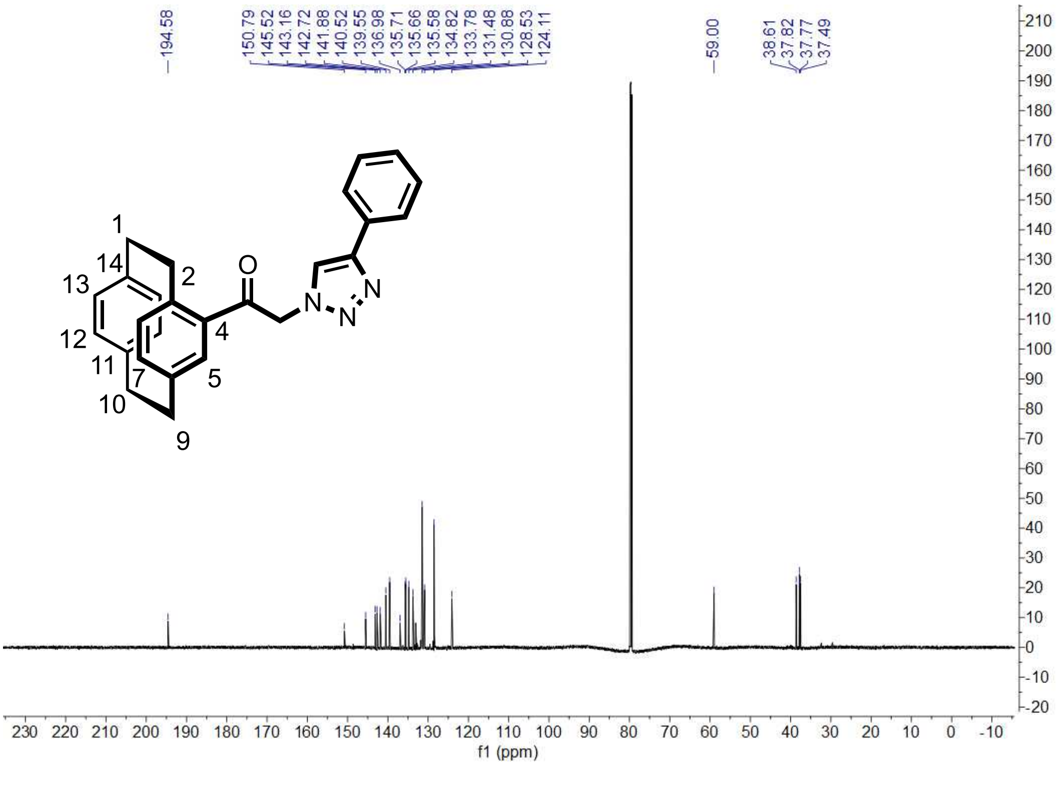

^{13}C NMR spectrum of **10** in $CDCl_3$

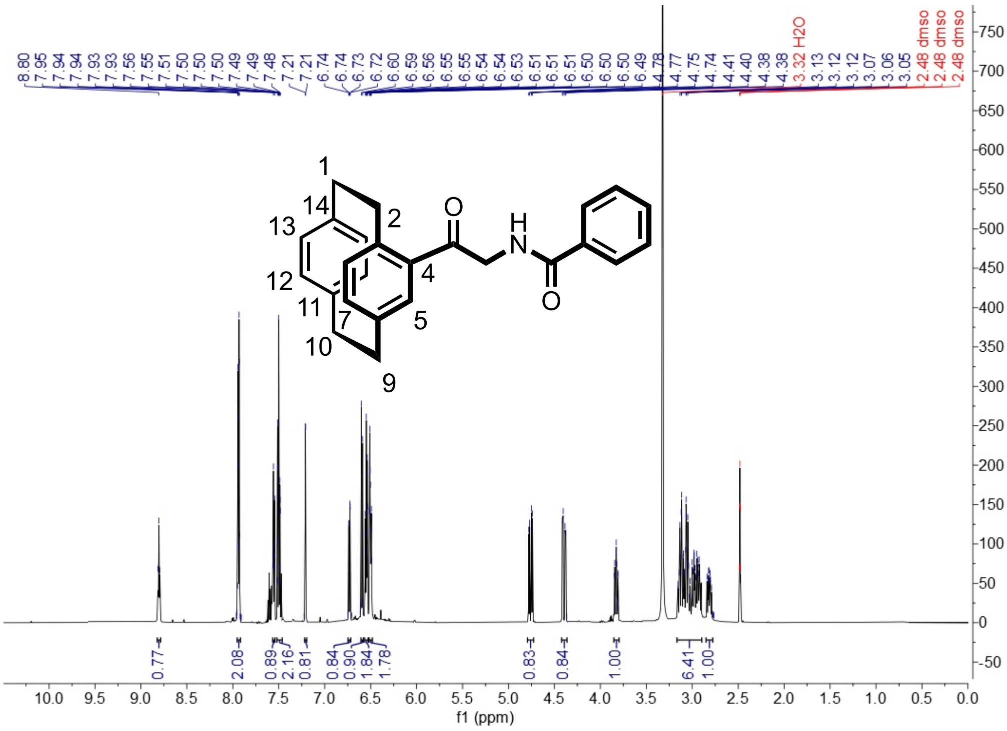

^{1}H NMR spectrum of **12a** in DMSO-D_6

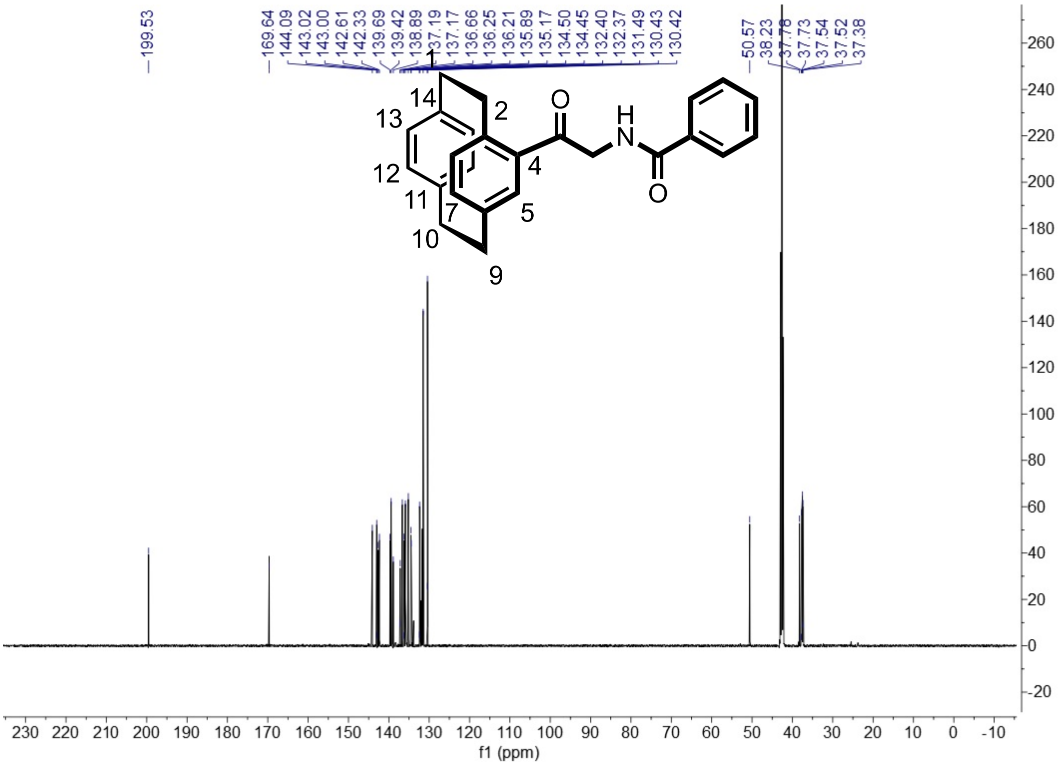

^{13}C NMR spectrum of **12a** in DMSO-D_6

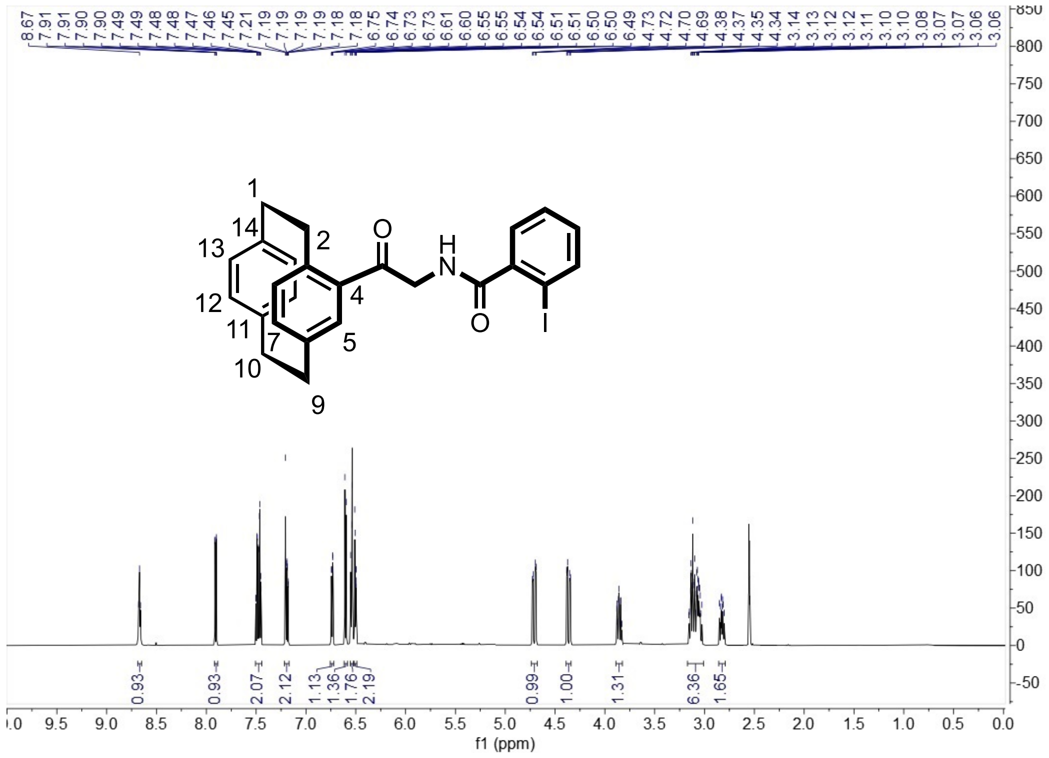

^{1}H NMR spectrum of **12b** in DMSO-D$_6$

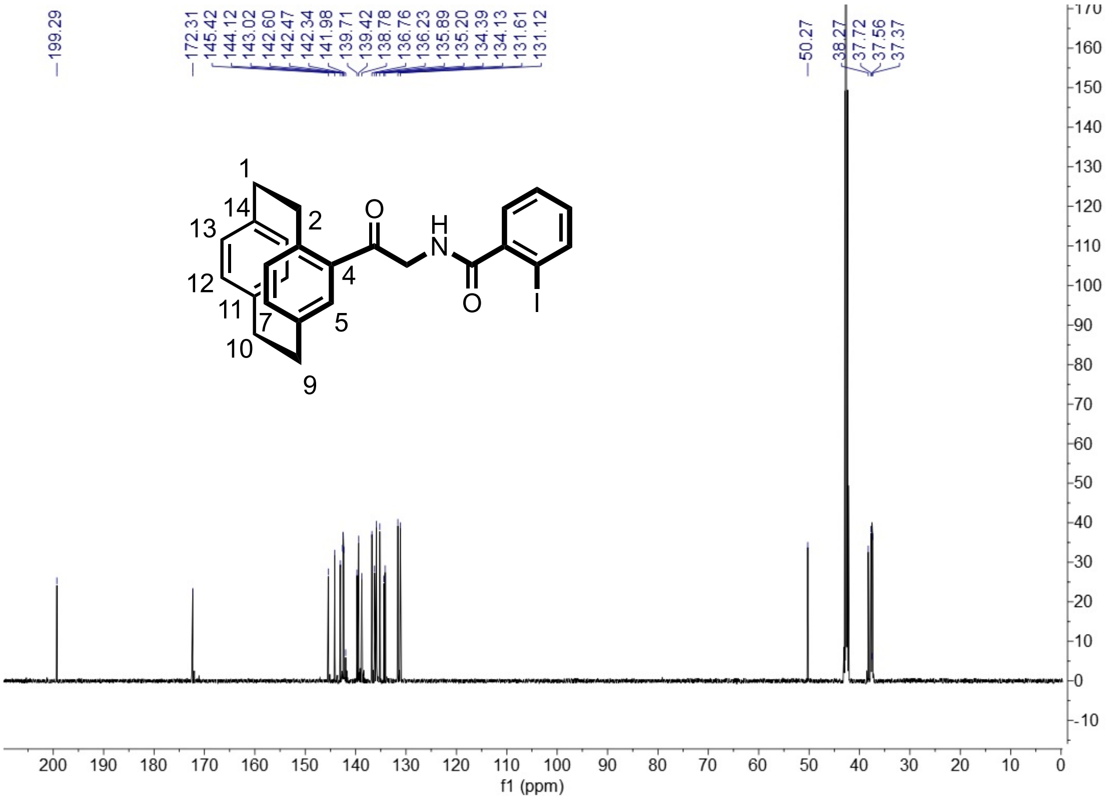

^{13}C NMR spectrum of **12b** in DMSO-D$_6$
